# Ischemic preconditioning protects the heart against ischemia-reperfusion injury in chronic kidney disease in both males and females

**DOI:** 10.1186/s13293-021-00392-1

**Published:** 2021-09-06

**Authors:** Márta Sárközy, Fanni Magdolna Márványkövi, Gergő Szűcs, Zsuzsanna Z. A. Kovács, Márton R. Szabó, Renáta Gáspár, Andrea Siska, Bence Kővári, Gábor Cserni, Imre Földesi, Tamás Csont

**Affiliations:** 1 MEDICS Research Group, Department of Biochemistry, Albert Szent-Györgyi Medical School Interdisciplinary Center of Excellence, University of Szeged, Szeged, 6720 Hungary; 2 Department of Laboratory Medicine, Albert Szent-Györgyi Medical School, University of Szeged, Szeged, 6720 Hungary; 3 Department of Pathology, Albert Szent-Györgyi Medical School, University of Szeged, Szeged, 6720 Hungary

**Keywords:** Uremic cardiomyopathy, Cardioprotection, Left ventricular hypertrophy and fibrosis, Diastolic dysfunction, Chronic renal failure, Ischemic preconditioning, Infarct size, Myocardial function, Reperfusion-induced salvage kinase (RISK) pathway, Survivor activating factor enhancement (SAFE) pathway

## Abstract

**Background:**

Uremic cardiomyopathy is a common cardiovascular complication of chronic kidney disease (CKD) characterized by left ventricular hypertrophy (LVH) and fibrosis enhancing the susceptibility of the heart to acute myocardial infarction. In the early stages of CKD, approximately 60% of patients are women. We aimed to investigate the influence of sex on the severity of uremic cardiomyopathy and the infarct size-limiting effect of ischemic preconditioning (IPRE) in experimental CKD.

**Methods:**

CKD was induced by 5/6 nephrectomy in 9-week-old male and female Wistar rats. Two months later, serum and urine laboratory parameters were measured to verify the development of CKD. Transthoracic echocardiography was performed to assess cardiac function and morphology. Cardiomyocyte hypertrophy and fibrosis were measured by histology. Left ventricular expression of A- and B-type natriuretic peptides (ANP and BNP) were measured by qRT-PCR and circulating BNP level was measured by ELISA. In a subgroup of animals, hearts were perfused according to Langendorff and were subjected to 35 min global ischemia and 120 min reperfusion with or without IPRE (3 × 5 min I/R cycles applied before index ischemia). Then infarct size or phosphorylated and total forms of proteins related to the cardioprotective RISK (AKT, ERK1,2) and SAFE (STAT3) pathways were measured by Western blot.

**Results:**

The severity of CKD was similar in males and females. However, CKD males developed more severe LVH compared to females as assessed by echocardiography. Histology revealed cardiac fibrosis only in males in CKD. LV ANP expression was significantly increased due to CKD in both sexes, however, LV BNP and circulating BNP levels failed to significantly increase in CKD. In both sexes, IPRE significantly decreased the infarct size in both the sham-operated and CKD groups. IPRE significantly increased the phospho-STAT3/STAT3 ratio in sham-operated but not in CKD animals in both sexes. There were no significant differences in phospho-AKT/AKT and phospho-ERK1,2/ERK1,2 ratios between the groups.

**Conclusion:**

The infarct size-limiting effect of IPRE was preserved in both sexes in CKD despite the more severe uremic cardiomyopathy in male CKD rats. Further research is needed to identify crucial molecular mechanisms in the cardioprotective effect of IPRE in CKD.

**Supplementary Information:**

The online version contains supplementary material available at 10.1186/s13293-021-00392-1.

## Highlights

1. There was no difference in the severity of chronic kidney disease (CKD) between male and female rats based on serum urea and creatinine levels as well as creatinine clearance.
2. As compared to females, males developed a more severe uremic cardiomyopathy characterized by left ventricular hypertrophy and fibrosis in CKD based on echocardiography and histology.
3. Following ischemia/reperfusion, infarct size was significantly smaller in females than in males, both in the sham-operated and CKD groups.
4. The infarct size-limiting effect of ischemic preconditioning (IPRE) was preserved in both sexes in CKD despite the more severe uremic cardiomyopathy in male CKD rats.
5. IPRE significantly increased the phospho-STAT3/STAT3 ratio in sham-operated, but not in CKD animals in both sexes.

**Supplementary Information:**

The online version contains supplementary material available at 10.1186/s13293-021-00392-1.

## Introduction

Chronic kidney disease (CKD) is one of the most rapidly growing non-communicable diseases and an important contributor to morbidity and mortality worldwide [1]. CKD is defined as abnormal renal structure and/or function (glomerular filtration rate [GFR] < 60 mL/min/1.73 m^2^) present for at least 3 months in patients [2]. In the general population, the global prevalence of CKD varies between 7 and 12% and is continuously increasing due to the growing incidence of its most common primary causes, including hypertension and diabetes mellitus [3]. The age-standardized global prevalence of early CKD stages (G1–G3, GFR > 30 mL/min/1.73 m^2^) is higher in women than in men [4, 5]. Notably, a higher CKD prevalence among women was found not only for CKD stages with decreased GFR but also for albuminuria with normal GFR [1]. Mortality is higher among men in all stages of predialysis CKD, whereas mortality among patients on renal replacement therapy is similar for men and women [4, 5].

CKD and end-stage renal disease (ESRD) patients have a 5- to 10-fold higher risk for developing cardiovascular diseases (CVDs) compared to the age-matched control population [6]. Large population studies have reported that all stages of CKD predispose to premature death, mainly from cardiovascular diseases (CVDs), and this is not restricted to ESRD [7]. Interestingly, the incidence of cardiovascular mortality is much higher in CKD patients at stages G2 and G3 than in ESRD patients [6, 8]. Uremic cardiomyopathy (i.e., type 4 cardiorenal syndrome) is defined as the structural, functional, and electrophysiological remodeling of the heart in CKD [9, 10]. It is characterized by left ventricular hypertrophy (LVH) and fibrosis, diastolic and systolic dysfunction, capillary rarefaction, and enhanced susceptibility to further injuries, including acute myocardial infarction (AMI) and arrhythmias [9]. Epidemiological and imaging studies proved that the primary manifestation of uremic cardiomyopathy is LVH and its prevalence increases with the progression of CKD (stage G3: 31%, G4: 50%, G5 and ESRD: 90%, respectively) [11–13]. The severity and persistence of LVH are strongly associated with cardiovascular events and mortality risk in CKD and ESRD patients [6]. Macrovascular disease seems to be more important in the early stages of CKD, and microvascular injury could play a major role in the late stages of CKD [7]. Therefore, AMI is a common cause of death in the early stages of CKD. In contrast, ESRD patients are more prone to sudden cardiac death due to arrhythmias and heart failure related to LVH, coronary calcification, and electrolyte disturbances [7].

One of the most powerful endogenous adaptive mechanisms of the heart is ischemic preconditioning (IPRE), in which brief cycles of myocardial ischemia and reperfusion periods significantly enhances the ability of the heart to withstand a subsequent prolonged ischemic injury (i.e., AMI) [14]. Preinfarction angina, warm-up phenomenon, and transluminal coronary angioplasty are considered clinical equivalents of IPRE [14]. Although IPRE confers remarkable cardioprotection in a variety of species [15, 16], including humans [17–19], we and others have shown in pre-clinical and clinical studies that its effectiveness is attenuated by age [14] and some co-morbidities, such as hypercholesterolemia [18, 20, 21] and diabetes mellitus [22–24]. However, some pre-clinical studies on CKD using male animals suggest that despite the complex systemic metabolic changes in CKD, cardioprotection by IPRE is still preserved. Byrne et al*.* reported that IPRE confers its cardioprotective effect via the RISK and SAFE pathways after 4 weeks of 5/6 nephrectomy or adenine-enriched diet-induced subacute renal failure in male rats [25]. However, experimental models of short-term renal failure may not correctly reflect the clinical situation because CKD frequently remains undiagnosed for a long time [26, 27]. Our research group found that IPRE still reduces the infarct size in prolonged uremia in male rats 30 weeks after 5/6 nephrectomy [28]. Nevertheless, so far, there is no experimental data available on ischemia/reperfusion (I/R) injury or the effects of IPRE in CKD in females. Therefore, we aimed to compare the severity of I/R injury and the potential cardioprotective effects of IPRE in CKD in male and female rats.

## Materials and methods

This investigation conformed to the National Institutes of Health Guide for the Care and Use of Laboratory Animals (NIH Publication No. 85-23, Revised 1996) and was approved by the Animal Research Ethics Committee of Csongrád County (XV.1181/2013-2018) and the University of Szeged in Hungary. All institutional and national guidelines for the care and use of laboratory animals were followed.

### Animals

A total of 160 age-matched female (*n* = 110, 9 weeks old, 180–200 g) and male (*n* = 86, 9-weeks old, 300–350 g) Wistar rats were used in this study. A total of 90 animals (46 males and 44 females) underwent a sham operation, and a total of 106 animals (40 males and 66 females) received 5/6 nephrectomy to induce CKD. After the operations, 4 animals (2 males and 2 females) died in the CKD groups. Animals were housed in pairs in individually ventilated cages (Sealsafe IVC system, Italy) and were maintained in a temperature-controlled room with 12-h:12-h light/dark cycles throughout the study. Standard rat chow and tap water were supplied ad libitum.

### Experimental setup

**Fig. 1:**
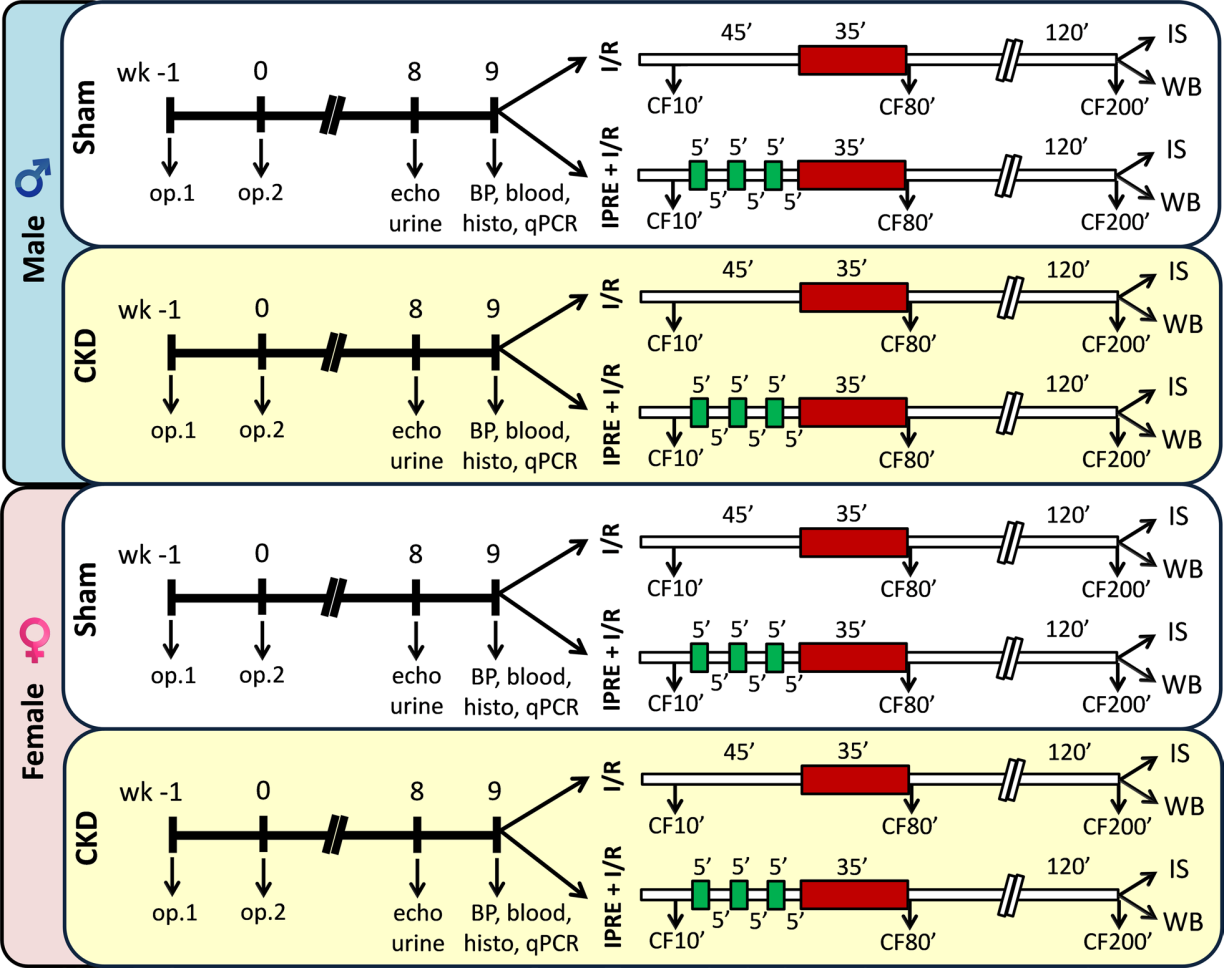
Protocol figure. Male and female Wistar rats underwent sham operation or 5/6 nephrectomy in 2 phases to induce chronic kidney disease (CKD). First, 2/3 of the left kidney was ligated and excised (Op1). One week later, the right kidney was removed (Op2). Corresponding time-matched sham operations were performed in the sham groups. At week 8, cardiac morphology and function were assessed by transthoracic echocardiography (echo) in a subgroup of animals. In this week, a subgroup of the animals was placed for 24 h into metabolic cages to collect urine for determination of urine creatinine and protein levels. At week 9, rats were anesthetized, and blood was collected from the thoracic aorta to measure serum urea and creatinine levels (blood). In this week, blood pressure was also measured in a subgroup of animals (BP). Hearts were then an isolated used for histology (histo) and qRT-PCR (qPCR) measurements or perfused according to Langendorff, the perfused hearts were subjected to global ischemia/reperfusion (I/R) with or without ischemic preconditioning protocol (IPRE, 3 × 5 min I/R cycles). Coronary flow (CF) was measured at the 10th, 80th, and 200th minutes of the perfusion protocol (CF10', CF80', and CF200', respectively). At the end of the perfusion protocol, hearts were collected for infarct size (IS) or Western blot (WB) measurements

Experimental CKD was induced by 5/6 nephrectomy. Animals underwent sham operation or 5/6 nephrectomy in two phases. After the operations, both groups were followed up for 9 weeks (Fig. 1). At week 8, cardiac morphology and function were assessed by transthoracic echocardiography in a subgroup of animals (*n* = 26 males [15 sham and 11 CKD] and 23 females [10 sham and 13 CKD]), (Fig. 1). Moreover, a subgroup of animals (*n* = 25 males [11 sham and 14 CKD] and *n* = 22 females [10 sham and 12 CKD]) was placed into metabolic cages at week 8 for 24 h to estimate creatinine clearance and measure urine creatinine and protein levels (Fig. 1). At week 9, blood was collected from the thoracic aorta (*n* = 27 males [14 sham and 13 CKD] and *n* = 26 females [12 sham and 14 CKD]), then serum urea, creatinine, ion levels, and plasma level of B-type natriuretic peptide (BNP) were measured. At the end of the follow-up period at week 9, hearts were isolated, then the blood was washed out in Krebs–Henseleit solution and hearts were used for histology and qRT-PCR measurements or perfused ex vivo by oxygenated Krebs–Henseleit solution, according to Langendorff. In the case of ex vivo perfusions, the menstrual cycle of the female rats was determined 12 h before the termination. To assess the cardioprotective effect of IPRE of sham-operated and CKD animals, the perfused hearts were subjected to global ischemia/reperfusion (I/R) with or without ischemic preconditioning protocol (IPRE) (Fig. 1). Coronary flow (CF) was measured (i) 10 min after heart cannulation (CF10’), (ii) immediately after the global ischemia in the 80th minute of the perfusion protocol (CF80’), and (iii) at the end of the reperfusion in the 200th minute of the protocol (CF200’) (Fig. 1). At the end of the appropriate perfusion protocol, the infarcted area was delineated using the triphenyl-tetrazolium chloride (TTC) staining method in a subgroup of animals (*n* = 43 in males and 57 in females). In another subgroup of animals, right and left ventricles were separated at the end of the perfusion protocols, and the LV samples were prepared for biochemical measurements (*n* = 24 males and *n* = 28 females) (Fig. 1). In this subgroup, left ventricular expression of phosphorylated and total proteins of STAT3, AKT, and ERK-1,2 were measured by Western blot technique (Fig. 1).

### 5/6 Nephrectomy

Sham operation and 5/6 nephrectomy were performed in two phases as described previously [28, 29] (Fig. 1). Anesthesia was induced by intraperitoneal injection of pentobarbital sodium (Euthasol; 40 mg/kg; Produlab Pharma b.v., Raamsdonksveer, The Netherlands). At the first operation, two pieces of sutures (5–0 Mersilk; Ethicon, Somerville, NJ) were placed around both poles of the left kidney, approximately at the 1/3 position. Then the sutures were gently ligated around the kidney. 1/3 of the kidney was excised right beyond the ligatures on both ends. Accidental bleeding was alleviated by thermal cauterization. One week after the first operation, animals were anesthetized, and the right kidney was freed from the surrounding adipose tissue and the renal capsule. It was then gently pulled out of the incision. The adrenal gland was gently freed and was placed back into the abdominal cavity. The renal blood vessels and the ureter were ligated, and the whole right kidney was removed. During sham operations, renal capsules were removed. After the surgeries, the incision was closed with running sutures and povidone–iodine (Betadine solution, 100 mg povidone–iodine complex/mL, Egis Zrt, Budapest, Hungary) was applied on the surface of the skin. As a postoperative medication, nalbuphine hydrochloride was administered for 4 days (*sc.* 0.3 mg/kg Nalbufin 10 mg/mL injection; TEVA Zrt, Debrecen, Hungary, twice in the first two postoperative days and once in the third and fourth postoperative days). Enrofloxacin antibiotics (Enroxil 75 mg tablets solved in drinking water [3.5 mg/L]; Krka, Slovenia) were administered in tap water for 4 days after both surgeries.

### Transthoracic echocardiography

Cardiac morphology and function were assessed by transthoracic echocardiography at week 8 as described previously [30–32] (Fig. 1). Briefly, rats were anesthetized with 2% isoflurane (Forane, AESICA, Queenborough Limited Kent, UK). Then, the chest was shaved, and the rat was placed in a supine position onto a heating pad. Two-dimensional, M-mode, Doppler, tissue Doppler and 4 chamber-view echocardiographic examinations were performed in accordance with the criteria of the American Society of Echocardiography with a Vivid IQ ultrasound system (General Electric Medical Systems) using a phased array 5.0–11 MHz transducer (GE 12S-RS probe). Data of three consecutive heart cycles were analyzed (EchoPac Dimension software; General Electric Medical Systems) by an experienced investigator in a blinded manner. The mean values of three measurements were calculated and used for statistical evaluation.

### Serum and urine laboratory parameters

At week 8, a subgroup of animals was placed into metabolic cages (Tecniplast, Italy) for 24 h to collect urine for the measurement of creatinine and protein levels. Urine creatinine and urine protein levels were measured by standard laboratory methods as described previously [28, 29] to verify the development of CKD (Fig. 1).

Blood was collected from the thoracic aorta at week 9 to measure serum urea (carbamide) and creatinine levels to verify the development of CKD. Serum urea and creatinine levels were quantified by kinetic UV method using urease and glutamate dehydrogenase enzymes and Jaffe method, respectively [28, 29] (Fig. 1). The reagents and the platform analyzers were from Roche Diagnostics (Mannheim, Germany).

Creatinine clearance, an indicator of renal function, was calculated according to the standard formula (urine creatinine concentration [μM] × urine volume for 24 h [mL])/(serum creatinine concentration [μM] × 24 × 60 min) as described previously [28, 29] (Fig. 1). Urine volume and urine creatinine concentration were measured at week 8, and serum creatinine concentration was determined at week 9.

### Plasma BNP levels

Plasma BNP level was determined as a marker of myocardial stretch in CKD at week 9 (*n* = 6–7, Fig. 1). The levels of BNP in the blood plasma of sham-operated and CKD rats were determined with enzyme-linked immunosorbent assay (ELISA) kits recognizing rat peptides (Sigma-Aldrich, USA, #RAB0386) in accordance with the manufacturer’s instructions.

### Blood pressure measurement

To measure arterial blood pressure, a 1.6 F, polyamide, pressure catheter (Scisense Systems Inc., London, ON, Canada) was inserted into the left femoral artery at week 9 under anesthesia (Euthasol; 40 mg/kg; Produlab Pharma b.v., Raamsdonksveer, The Netherlands) in a separated subgroup of animals (*n* = 6–11) as described previously [29, 33] (Fig. 1). Blood pressure measurements were performed between 09:00 and 14:00 hours.

### Histological measurements on hematoxylin–eosin and picrosirius red/fast green-stained sections

In a subgroup of the animals (*n* = 6–9), 5-μm-thick sections from formalin-fixed paraffin-embedded tissue blocks taken transversely from the subvalvular areas of the left ventricles were stained with hematoxylin–eosin (HE) or picrosirius red/fast green (PSFG) as described previously [29–31] (Fig. 1). Histological sections were scanned with a Pannoramic Midi II scanner (3D-Histech, Hungary) and digital images at the magnification of × 10, × 40 and × 100 were captured. To verify the development of left ventricular hypertrophy, cardiomyocyte perimeter was measured in 100 selected, longitudinally oriented, mono-nucleated cardiomyocytes on digital images from a single left ventricular transverse slide. [29–31].

Cardiac fibrosis was assessed on PSFG slides with an in-house developed program as described previously [29–31]. Briefly, this program determines the proportion of red pixels of heart sections using two simple color filters. For each red–green–blue (RGB) pixel, the program calculates the color of the pixel in hue–saturation–luminance (HSL) color space. The first filter is used for detecting red portions of the image. The second filter excludes any white (empty) or light grey (residual dirt on the slide) pixel from further processing using a simple RGB threshold. In this way, the program groups each pixel into one of two sets: pixels considered red and pixels considered green but neither white, nor grey. Red pixels in the first set represent collagen content and fibrosis. Green pixels in the second set correspond to cardiac muscle. The mean values of 10 representative images were calculated and used for statistical evaluation in the case of each left ventricular slide. Medium-size vessels and their perivascular connective tissue sheet, the subepicardial and subendocardial areas were avoided as much as possible.

### qRT-PCR measurements

Quantitative RT-PCR was performed with gene-specific primers to monitor left ventricular mRNA expression at week 9 [29]. Total RNA was isolated from left ventricles (*n* = 7–11) with 5:1 mixture of Biozol total RNA extraction reagent (Bioflux, China) and chloroform (Molar Chemicals, Hungary). The RNA containing aqueous phase was further precipitated with isopropanol (Molar Chemicals, Hungary). RNA concentration and purity were determined by spectrophotometric measurement with NanoDrop One (Thermo Scientific, Waltham, USA). Then 100 μg of total RNA was reverse transcribed using iScript™ cDNA Synthesis Kit (BioRad Laboratories Inc., USA). Specific primers (*Nppa*: A-type natriuretic peptide, #qRnoCED0006216 and *Nppb*: B-type natriuretic peptide, #qRnoCED0001541) and SsoAdvanced™ Universal SYBR® Green Supermix (BioRad Laboratories Inc., USA) were used according to the manufacturer’s instructions. Peptidyl-prolyl isomerase A (*Ppia*, forward primer sequence: tgctggaccaaacacaaatg, reverse primer sequence: caccttcccaaagaccacat) was used as a house keeping control gene for normalization.

### Vaginal smear and menstrual cycle

The menstrual cycle of the female animals was tested before heart perfusion protocols because the fluctuation of estrogen and progesterone levels may influence the infarct size and protein expression levels. We aimed to select the female rats in the di-estrus phase in which the hormone levels are the lowest. The menstrual cycle was determined 12 h before the heart perfusions. For vaginal smear collection, a cotton earbud was used after physiologic saline slosh. Vaginal smear was put on glass slides, stained by Giemsa solution, and fixed with a mixture of 20% alcohol and diethyl-ether in a 1:9 ratio. The slides were examined by light microscopy under × 40 magnification. The amount of vaginal epithelial cells, their morphology, and the presence of lymphocytes were evaluated.

### Ex vivo cardiac perfusions and ischemic preconditioning

At week 9, rats were anesthetized, and hearts were isolated and perfused at 37 °C according to Langendorff with oxygenated Krebs–Henseleit buffer as previously described [28, 34]. Hearts from the sham-operated and the CKD groups were further divided into two subgroups and subjected to either a non-conditioning or preconditioning perfusion protocol, respectively (Fig. 1). Non-conditioned hearts were subjected to time-matched (45 min) aerobic perfusion followed by 35-min global I/R. Preconditioned hearts were subjected to 5 min of ischemia and 5 min reperfusion in 3 intermittent cycles before the 35 min of global index ischemia. Coronary flow was measured at the 10th, 80th, and 200th min of the perfusion protocol, respectively. At the end of the 2-h reperfusion, the hearts were weighed and used for infarct size or biochemical measurements. Body, lung, and kidney weights were also measured.

### Infarct size determination

After the end of reperfusion, in a subgroup of hearts, atria were removed, and the total ventricles were used to determine the infarcted area as described previously [34, 35]. Briefly, frozen ventricles were cut to 7–8 equal slices and placed into triphenyl-tetrazolium chloride solution (Sigma-Aldrich, Saint Louis, MO, USA) for 10 min at 37 °C followed by a 10 min formaldehyde fixation and phosphate buffer washing steps. As a result, survived areas were red-stained while the necrotic area remained pale. Digitalized images from the stained heart slices were evaluated with planimetry, and the amount of myocardial necrosis was expressed as infarct size/area at risk % as described previously [36].

### Western blot

At the end of the 2 h reperfusion, hearts were separated for left and right ventricles in a subgroup of animals. Left ventricles were used for standard Western blot measurements to investigate gene expression changes at the protein level in the case of phospho-AKT, AKT, phospho-ERK1,2, ERK1,2, phospho-STAT3, STAT3 with GAPDH loading background. Left ventricular tissue samples (*n* = 52) were homogenized with an ultrasonicator (UP100H Hielscher, Teltow, Germany) in Radio Immunoprecipitation Assay (RIPA) buffer (50 mM Tris–HCl (pH 8.0), 150 mM NaCl, 0.5% sodium deoxycholate, 5 mM ethylenediamine tetra-acetic acid (EDTA), 0.1% sodium dodecyl sulphate, 1% NP-40 (Cell Signaling, Carlsbad, CA, USA) supplemented with protease inhibitor cocktail and phosphatase inhibitors phenylmethylsulfonyl fluoride (PMSF) and sodium fluoride (NaF, Sigma, Saint Louis, USA). The crude homogenates were centrifuged at 15,000×*g* for 30 min at 4 °C. After quantification of protein concentrations of the supernatants using BCA Protein Assay Kit (Pierce, Rockford, IL, USA), 25 μg of reduced and denatured protein was loaded. Then sodium dodecyl sulfate–polyacrylamide gel electrophoresis (SDS-PAGE) was performed (10% gel, 90 V, 2 h in case of AKT, ERK1,2, STAT3), which was followed by transfer of proteins onto a nitrocellulose membrane (20% methanol, 35 V, 2 h in case of AKT, ERK1,2, STAT3). The efficacy of transfer was checked using Ponceau staining. The membranes were cut horizontally into parts corresponding to the molecular weights of AKT, ERK1,2, STAT3, and GAPDH and were blocked for 1 h in 5% (w/v) bovine serum albumin (BSA) or milk at room temperature and then incubated with primary antibodies (Cell Signaling, Carlsbad, CA, USA) in the concentrations of 1:1000 against phospho-AKT (Ser473, #4060), AKT (#9272), phospho-ERK1,2 (Tr202/Tyr204, #9101 S), ERK1,2 (#9102), phospho-STAT3 (Ser727; #9134) overnight, 4 °C, 5% BSA), and 1:5000 against GAPDH (#2118, overnight, 4 °C, 1% BSA). Then, the membranes were incubated with IRDye® 800CW Goat Anti-Rabbit and IRDye® 680RD Goat Anti-Mouse secondary antibody (Li-Cor) for 1 h at room temperature in 5% BSA. Fluorescent signals were detected by Odyssey CLx, and digital images were analyzed and evaluated by Quantity One Software [37].

### Statistical analysis

Statistical analysis was performed using Sigmaplot 12.0 for Windows (Systat Sofware Inc). All values are presented as mean ± SEM. Specific sample numbers used for measurements are described in the corresponding figure legend. Baseline and different follow-up data, including serum metabolite and ion concentrations, plasma BNP level, and echocardiographic, histologic and qRT-PCR data were compared using two-way analysis of variance (ANOVA). Infarct size and Western blot measurement data were compared using three-way ANOVA. Holm–Sidak test was used as a post hoc test*.*

## Results

### Males and females develop a similar severity of chronic kidney disease based on routine laboratory parameters

**Fig. 2:**
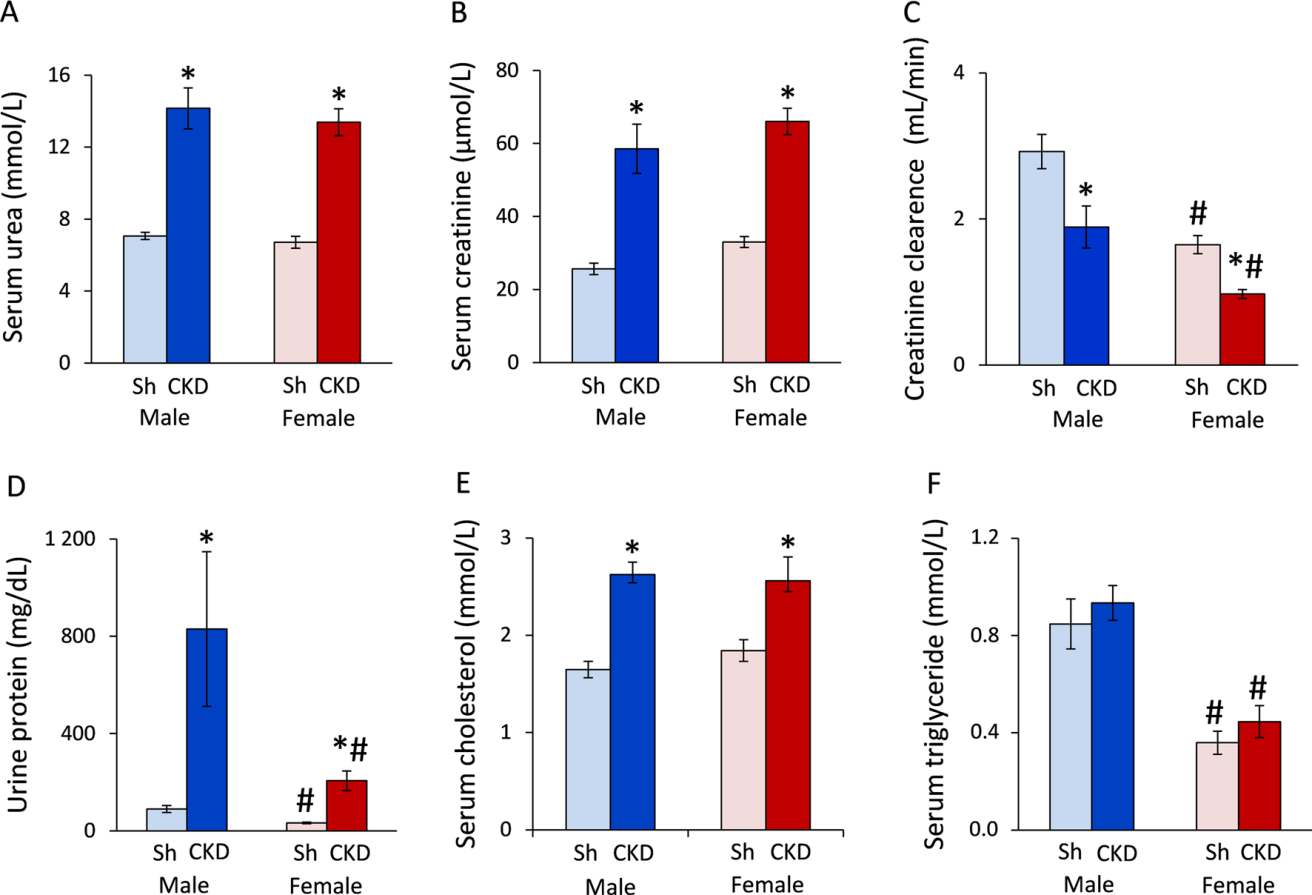
The effects of sex and CKD on laboratory markers. **A** Serum urea concentration; **B** serum creatinine concentration; **C** creatinine clearance was calculated from urine volume, urine and serum creatinine concentrations according to the formula: urine creatinine concentration [μM] × urine volume for 24 h [mL])/(serum creatinine concentration [μM] × 24 × 60 min¥; **D** urine protein concentration; **E** serum cholesterol concentration, and **F** serum triglyceride concentration. Urine volume and urine creatinine concentration were measured at week 8, and serum creatinine concentration was determined at week 9. Values are means ± SEM, *n* = 12–14 for serum parameters (sham male: *n* = 13, CKD male: *n* = 14, sham female: *n* = 12, and CKD female: *n* = 14), and *n* = 10–14 for urine parameters and creatinine clearance (sham male: *n* = 11, CKD male: *n* = 14, sham female: *n* = 10, and CKD female: *n* = 12), *p < 0.05, CKD vs. sham-operated groups, #*p* < 0.05, females vs. males, *p*-values refer to two-way ANOVA (Holm–Sidak post hoc test)

To verify the development of CKD induced by 5/6-nephrectomy, concentrations of several serum and urine metabolites were measured at week 9. The serum urea and creatinine levels were similar in the sham-operated males and females (*p* = 0.388 and *p* = 0.709, Fig. 2A and B, respectively). The serum urea levels showed a significant increase to 200% in both sexes in 5/6-nephrectomized rats compared to the sham-operated animals (*p < 0.001 in both sexes, Fig. 2A). The serum creatinine levels were significantly higher in male and female 5/6-nephrectomized rats (253%, *p < 0.001 and 217%, *p < 0.001, respectively) than in the sex-matched sham-operated animals (Fig. 2B). The creatinine clearance was significantly lower (57%) in the sham-operated females than in the sham-operated males (^#^*p* < 0.001), probably due to the smaller body weight and muscle mass of the females (Fig. 2C). Moreover, the creatinine clearance showed a significant decrease in male and female 5/6-nephrectomized rats (56%, *p < 0.001 and 59%, *p = 0.031, respectively) compared to the sex-matched sham-operated animals showing a similarly decreased renal function in both sexes (Fig. 2C). The urine protein concentration was significantly lower (36%) in the sham-operated females than males (^#^*p* = 0.010, Fig. 2D). Urine protein concentration was significantly increased in male and female 5/6-nephrectomized rats (to 930% and 640%, respectively, *p < 0.001 in both sexes) compared to their sex-matched controls (Fig. 2D). Serum cholesterol and triglyceride levels were measured as cardiovascular risk factors. Serum cholesterol levels were similar in the sham-operated animals (*p* = 0.352); however, serum triglyceride level was significantly lower in the sham-operated females than males (42%, ^#^*p* < 0.001) (Fig. 2E, F). Serum cholesterol levels were significantly elevated in male and female 5/6-nephrectomized rats (to 158% and 139%, respectively, *p < 0.001 in both sexes) compared to the sex-matched sham-operated animals (Fig. 1E). Serum triglyceride levels were significantly reduced (to 48%) in female 5/6-nephrectomized rats compared to 5/6-nephrectomized males (^#^*p* < 0.001, Fig. 2E). Lower serum triglyceride levels in females irrespective of CKD might refer to their reduced cardiovascular risk as compared to males.

### Uremic cardiomyopathy is more severe in males than in females

#### Males develop more severe LVH and diastolic dysfunction in CKD than females

**Fig. 3:**
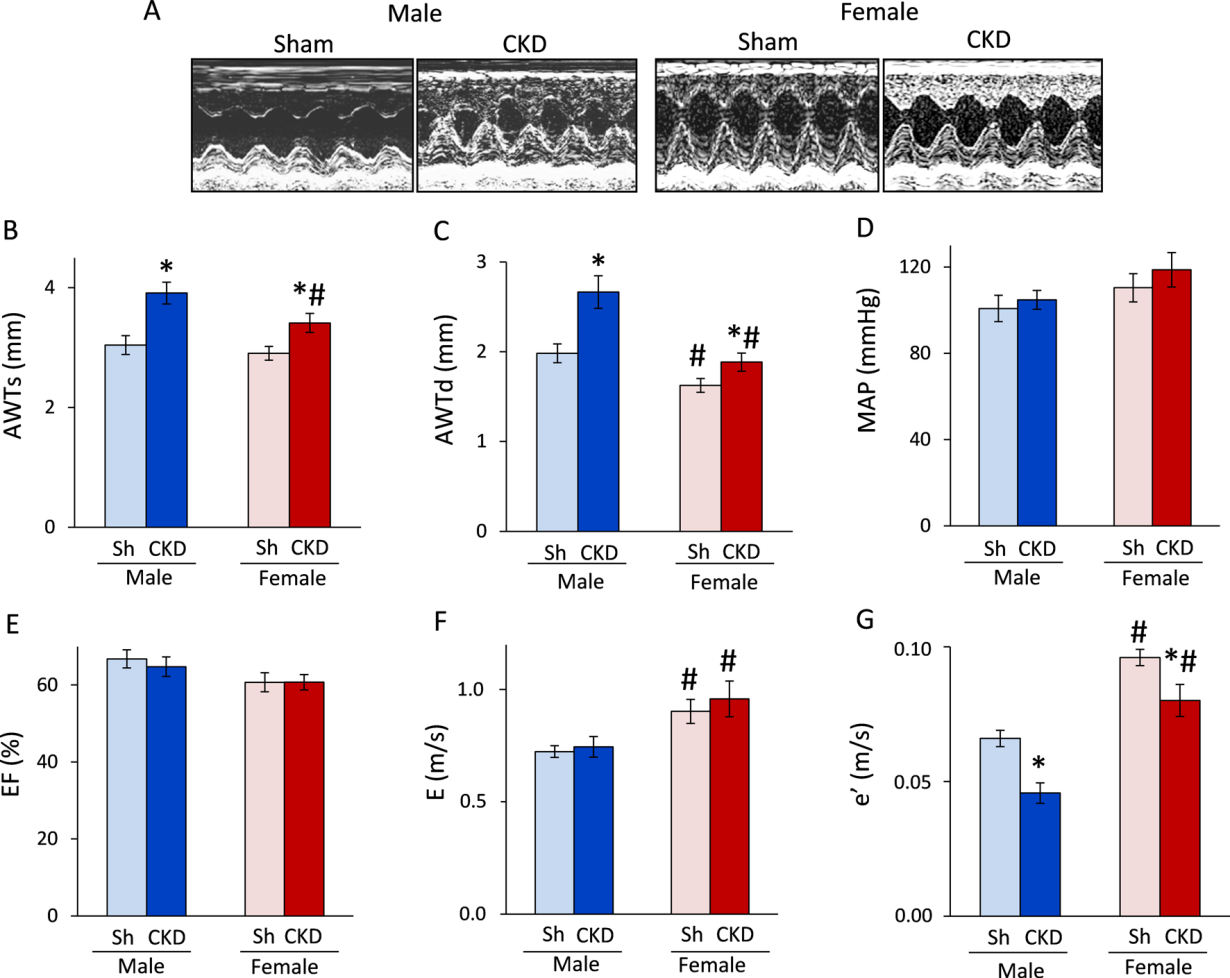
The effects of sex and CKD on echocardiographic parameters and blood pressure. **A** Representative M-mode images; **B** anterior wall thicknesses in systole (AWTs); **C** anterior wall thicknesses in diastole (AWTd); **D** mean arterial blood pressure (MAP); **E** ejection fraction (EF); **F** *E*/*e*′ ratio; **G** isovolumic relaxation time (IVRT) measured at week 9. Values are means ± SEM, *n* = 10–15 for echocardiography (sham male: *n* = 15, CKD male: *n* = 11, sham female: *n* = 10, and CKD female: *n* = 13) and *n* = 6–11 for blood pressure measurement (sham male: *n* = 10, CKD male: *n* = 11, sham female: *n* = 6, and CKD female: *n* = 8), *p < 0.05, CKD vs. sham-operated groups, #*p* < 0.05, females vs. males *p*-values refer to two-way ANOVA (Holm–Sidak post hoc test)

Transthoracic echocardiography was performed at week 8 to investigate whether CKD development leads to similar myocardial morphologic and functional alterations in both sexes. Most of the left ventricular systolic and diastolic wall thicknesses were not significantly different between males and females in the sham-operated groups (Fig. 3A–C, Table 1). Among wall thicknesses, the diastolic anterior and septal wall thickness were significantly decreased (to 82% and 84%, respectively) in the sham-operated females compared to sham-operated males (^#^*p* < 0.001, Fig. 3 and Table 1) due to their smaller heart size. Notably, the systolic septal and diastolic inferior wall thicknesses showed a trend toward a decrease in the sham-operated females compared to sham-operated males (Table 1). The left ventricular end-diastolic and end-systolic diameters were significantly reduced (to 92% and 61%, respectively) in sham-operated females compared to sham-operated males, probably corresponding to the smaller body and heart size of females (Table 1). In response to CKD, systolic and diastolic anterior wall thicknesses were significantly increased in both sexes (to 129%, and 134% in males and 117% and 116% in females, respectively) compared to the sex-matched sham-operated animals (*p < 0.001 for both sexes and walls, Fig. 3B, C). In CKD males, diastolic inferior, posterior, and septal, and systolic septal wall thicknesses were significantly increased compared to those of sham-operated males (to 130%, 128%, 124%, and 122%, respectively) (Table 1). In contrast, CKD females showed a smaller relative increase in fewer wall thicknesses than CKD males (Fig. 3A–C and Table 1). In CKD females, diastolic inferior, diastolic and systolic septal wall thicknesses were significantly increased compared to those of sham-operated females (to 118%, 111%, and 109%, respectively) (Table 1). Left ventricular end-diastolic and end-systolic diameters showed no significant difference in response to CKD in either sex. However, left ventricular end-systolic diameter showed a trend toward a decrease in males in response to CKD, probably due to the more severe LVH (Table 1). Left ventricular end-diastolic volume, stroke volume, and cardiac output were significantly decreased in CKD males as compared to sham-operated males (to 74%, 70%, and 70%, respectively) (Table 1). In contrast, these parameters were not changed significantly in CKD females when compared to sham-operated females (Table 1). Notably, cardiac output was significantly lower in sham-operated females than in sham-operated males, corresponding to the smaller heart size of females (Table 1). Interestingly, left ventricular end-systolic volume was significantly increased in CKD in females as compared to males, probably due to the less severe LVH in females. The heart rate, systolic, diastolic, and mean blood pressure were not different in response to CKD in both sexes (Table 1). However, sham-operated and CKD females had a 12–14% higher systolic and diastolic blood pressure than that of sham-operated and CKD males, respectively (Table 1). Mean blood pressure only showed a tendency to increase in sham-operated and CKD females compared to sham-operated and CKD males, respectively (*p* = 0.075, Fig. 3D). The main systolic functional parameter, ejection fraction, did not change significantly between the groups (*p* = 0.559, Fig. 3E). The *E*- and *e*’-velocities were measured by echocardiography to assess the diastolic function (Fig. 3F, G). The *E*- and *e*’-velocities were significantly increased in sham-operated and CKD females compared to sham-operated and CKD males, respectively (to 113% and 142% for *E*, and 130% and 160% for *e*’, respectively) (^#^*p* < 0.001 for both parameters, Fig. 3F, G). The *E* velocity did not change significantly in response to CKD in either sexes (*p* = 0.560, Fig. 3F). In contrast, *e*’ was significantly reduced in response to CKD in both sexes (to 70% in males and 83% in females, *p < 0.001, Fig. 3G), pointing out the presence of diastolic dysfunction in CKD.

**Table 1:** The effects of sex and CKD on various in vivo left ventricular morphological and cardiac functional parameters

Parameter (unit)	Male		Female		*p*-value	
	Sham	CKD	Sham	CKD	CKD vs. sham	Female vs. male
IWTs (mm)	3.07 ± 0.09	3.48 ± 0.21	3.31 ± 0.11	3.67 ± 0.14	0.051	0.373
IWTd (mm)	1.86 ± 0.18	2.42 ± 0.33*	1.72 ± 0.23	2.03 ± 0.09*	0.011	0.066
PWTs (mm)	3.00 ± 0.10	3.42 ± 0.24	3.24 ± 0.12	3.54 ± 0.18	0.089	0.602
PWTd (mm)	1.74 ± 0.05	2.23 ± 0.25*	1.82 ± 0.06	1.97 ± 0.14	0.038	0.203
SWTs (mm)	3.26 ± 0.11	3.98 ± 0.21*	3.32 ± 0.08	3.63 ± 0.12*	< 0.001	0.074
SWTd (mm)	2.02 ± 0.10	2.52 ± 0.18*	1.69 ± 0.08^#^	1.88 ± 0.07^#^	0.006	< 0.001
LVEDD (mm)	6.73 ± 0.17	6.50 ± 0.36	6.20 ± 0.14	6.41 ± 0.11	0.547	0.761
LVESD (mm)	3.51 ± 0.17	2.92 ± 0.37	2.15 ± 0.19^#^	2.07 ± 0.15^#^	0.182	0.011
Heart rate (1/min)	363 ± 6	347 ± 14	370 ± 12	379 ± 10	0.479	0.045
LVEDV (μL)	102 ± 6	75 ± 6*	89 ± 9	99 ± 6^#^	0.029	0.031
LVESV (μL)	33 ± 4	26 ± 3	36 ± 4	39 ± 3^#^	0.377	0.021
SV (μL)	70 ± 4	49 ± 5*	53 ± 6	60 ± 4	0.011	0.120
CO (mL/min)	25 ± 1	17 ± 2*	19 ± 2^#^	23 ± 1*	0.003	0.013
SBP (mmHg)	111 ± 6	120 ± 4	126 ± 6^#^	136 ± 8^#^	0.117	0.017
DBP (mmHg)	87 ± 5	94 ± 4	98 ± 7^#^	107 ± 8^#^	0.215	0.048

Transthoracic echocardiographic and blood pressure measurements were performed 8 and 9 weeks after 5/6 nephrectomy, respectively. Values are mean ± SEM, *n* = 10–15 for echocardiography (sham male: *n* = 15, CKD male: *n* = 11, sham female: *n* = 10, and CKD female: *n* = 13), and *n* = 6–11 for blood pressure measurement (sham male: *n* = 10, CKD male: *n* = 11, sham female: *n* = 6, and CKD female: *n* = 8), *p < 0.05, CKD vs. sham-operated groups, #*p* < 0.05, females vs. males; *p*-values refer to two-way ANOVA (Holm–Sidak post hoc test). *CKD* chronic kidney disease, *CO* cardiac output, *d* diastolic, *DBP* diastolic blood pressure, *E-wave* early ventricular filling velocity, *e'* e'-wave, mitral annulus velocity measured by tissue Doppler, *IWT* inferior wall thickness, *LVEDD* left ventricular end-diastolic diameter, *LVEDV* left ventricular end-diastolic volume, *LVESD* left ventricular end-systolic diameter, *LVESV* left ventricular end-systolic volume, *PWT* posterior wall thickness, *s* systolic, *SBP* systolic blood pressure, *SV* stroke volume, *SWT* septal wall thickness

#### Only males develop severe cardiac fibrosis in CKD

**Fig. 4:**
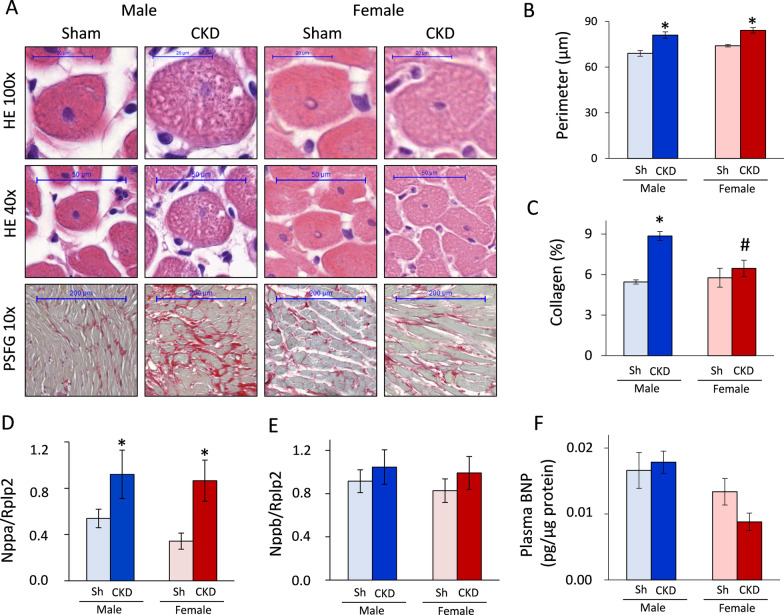
The effects of sex on cardiomyocyte hypertrophy, fibrosis and heart failure markers. **A** Representative hematoxylin–eosin (HE, × 40 and × 100) and picrosirius red/fast green-stained (PSFG, × 10) sections; **B** cardiomyocyte perimeter; **C** left ventricular collagen content; **D** left ventricular A-type natriuretic peptide (ANP) expression; **E** left ventricular B-type natriuretic peptide (BNP) expression; **F** circulating plasma BNP level measured at week 9. Values are means ± SEM, *n* = 6–9 for histology (sham male: *n* = 6, CKD male: *n* = 9, sham female: *n* = 6, and CKD female: *n* = 7), *n* = 7–11 for ANP and BNP expression (sham male: *n* = 8, CKD male: *n* = 8, sham female: *n* = 9, and CKD female: *n* = 11), and *n* = 6–7 for circulating BNP level (sham male: *n* = 7, CKD male: *n* = 6, sham female: *n* = 6, and CKD female: *n* = 6), *p < 0.05, CKD vs. sham-operated groups, #*p* < 0.05, females vs. males *p*-values refer to two-way ANOVA (Holm–Sidak post hoc test)

To verify the echocardiographic signs of cardiac remodeling in CKD, cardiomyocyte perimeter was measured on HE-stained slides, while collagen content was assessed on PSFG-stained slides (Fig. 4A). Both males and females had significantly increased cardiomyocyte perimeters in CKD as compared to the sex-matched sham-operated groups, proving the development of cardiomyocyte hypertrophy at the microscopic level (*p < 0.001, Fig. 4B). However, there was no significant sex-based difference in the cardiomyocyte perimeters within the CKD or sham-operated groups (*p* = 0.086 within sham groups and *p* = 0.219 within the CKD groups, Fig. 4B).

The male CKD group demonstrated significantly increased left ventricular collagen content as compared to the male sham-operated group, proving the development of cardiac fibrosis in CKD (*p < 0.001, Fig. 4). In contrast, there was no significant difference in the left ventricular collagen content between the female sham-operated and CKD groups, pointing out a less severe cardiac remodeling in females in CKD (*p* = 0.333, Fig. 4C). Notably, the left ventricular collagen content was significantly reduced in the female CKD group as compared to the male CKD group (^#^*p* = 0.001). There was no difference in the collagen content between males and females in the sham-operated groups (*p* = 0.671, Fig. 4C).

#### Increased left ventricular ANP expression in CKD in both sexes

Left ventricular ANP and BNP expressions as well as circulating BNP levels were measured as myocardial stretch markers. The left ventricular ANP expression was significantly increased in both sexes in CKD as compared to the sex-matched sham-operated groups (*p* = 0.018, Fig. 4D). There was no significant sex-based difference in the left ventricular ANP expression within the sham-operated or CKD groups (Fig. 4D). Interestingly, there were no significant differences in the left ventricular BNP expression and plasma BNP level between the groups (Fig. 4E, F). Notably, plasma BNP level in the female CKD group was half of the value of the male CKD group (*p* = 0.067, Fig. 4F), suggesting a less severe uremic cardiomyopathy in females.

### Effects of sex and CKD on ex vivo morphological and functional parameters

Morphological parameters including body weight, tibia length, heart weight, left ventricular weight, left kidney weight, and lungs’ weight, were measured at week 9 to investigate whether CKD influences ex vivo parameters similarly in both sexes. The body weight and tibia length of females were significantly lower both in sham-operated (55% and 89%, respectively) and CKD females (56% and 89%, respectively) compared to sham-operated and CKD males, respectively (Table 2). Both CKD males and females had significantly lower body weight (92% and 95%, respectively) and tibia length (98% and 97%, respectively) as compared to sham-operated males and females (Table 2), indicating the development of uremic cachexia of similar severity in both sexes. Heart weight, left ventricular weight, lungs’ weight, and kidney weights were significantly lower in females than in males irrespective of CKD due to the smaller body size of females (Table 2). In response to CKD, heart weight showed a trend toward an increase in both sexes compared to sham-operated animals (Table 2). Left ventricular weights were significantly increased in CKD both in males and females (to 109% and 114%, respectively) compared to sham-operated males and females, respectively, confirming the development of LVH (Table 2). Notably, lungs’ weight was slightly increased only in male CKD rats (to 111%), but not in female CKD rats, compared to sham-operated sex-matched animals, indicating the development of mild pulmonary congestion and more severe uremic cardiomyopathy in males than in females. Interestingly, the whole left kidney’s weight in the sham-operated males and females were markedly higher than the weight of remaining one-third of the left kidney in the CKD males and females (136% and 120%, respectively), suggesting a frank compensatory renal hypertrophy in the CKD animals (Table 2).

**Table 2:** The effects of sex and CKD on ex vivo heart weights, tibia length, and basal coronary flow

Parameter (unit)	Male		Female		*p*-value	
	Sham	CKD	Sham	CKD	CKD vs. sham	Female vs. male
Body weight (g)	488 ± 8	451 ± 7*	268 ± 4^#^	254 ± 3*^#^	< 0.001	< 0.001
Tibia length (cm)	4.27 ± 0.03	4.18 ± 0.06*	3.82 ± 0.04^#^	3.73 ± 0.03*^#^	0.049	< 0.001
Heart weight (mg)	2450 ± 68	2541 ± 63	1476 ± 34^#^	1536 ± 32^#^	0.210	< 0.001
LV weight (mg)	1319 ± 34	1434 ± 49*	900 ± 33^#^	1023 ± 34*^#^	0.005	< 0.001
CF10' (mL/min)	17.5 ± 0.8	17.4 ± 1.0	11.0 ± 0.4^#^	12.1 ± 0.4^#^	0.720	< 0.001
Kidney weight (mg)	1330 ± 43	1809 ± 79	989 ± 50^#^	1189 ± 75	N/A	< 0.001
Lung weight (mg)	1596 ± 55	1765 ± 67	1508 ± 36^#^	1464 ± 37^#^	0.242	< 0.001

Values are mean ± SEM, *n* = 30–55 for body weight, heart weight and CF10’ (sham male: *n* = 35, CKD male: *n* = 31, sham female: *n* = 30, and CKD female: *n* = 55), and *n* = 13–19 for tibia length, LV weight, kidney weight and lung weight (sham male: *n* = 19, CKD male: *n* = 13, sham female: *n* = 18, and CKD female: *n* = 18), *p < 0.05, CKD vs. sham-operated groups, #*p* < 0.05, females vs. males, respectively, *p*-values refer to two-way ANOVA (Holm–Sidak post hoc test). *CF10'* coronary flow collected at the 10th minute or perfusion protocol, *CKD* chronic kidney disease, *LV* left ventricular

### Selection of the female rats before heart perfusion protocols based on vaginal smear investigation

**Fig. 5:**
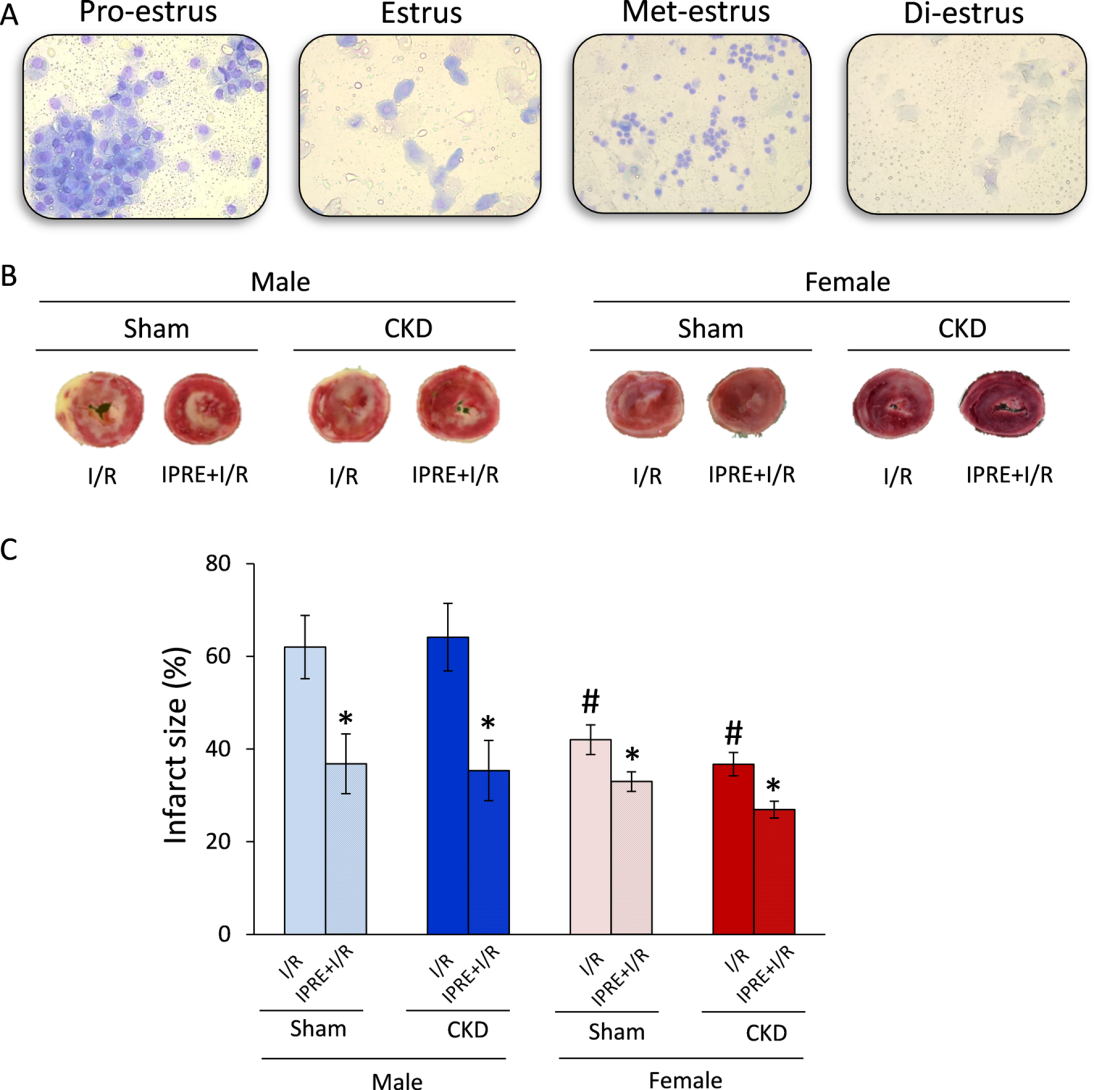
The effects of sex and CKD on the cardioprotection conferred by IPRE. **A** Representative images of the estrus cycle in female rats; **B** representative images of myocardial infarction stained by triphenyl-tetrazolium chloride (TTC), and **C** infarct size (IS). Values are means ± SEM, *n* = 8–8 in males (in every subgroup) and *n* = 9–19 in females (sham I/R: *n* = 9, sham IPRE + I/R: *n* = 10, CKD I/R: *n* = 19, and CKD IPRE + I/R: *n* = 19) for IS, *p < 0.05, IPRE + I/R vs. I/R subgroups, #*p* < 0.05, females vs. males, *p*-values refer to three-way ANOVA (Holm–Sidak post hoc test). *CKD* chronic kidney disease, *IPRE* ischemic preconditioning, *I*/*R* ischemia/reperfusion

The fluctuation of estrogen and progesterone levels during the menstrual cycle may acutely influence the infarct size and cardiac protein expression patterns. Therefore, female rats were selected 12 h before the heart perfusion protocols according to their menstrual cycle as assessed by vaginal smear investigation. In the pro-estrus phase, many nucleated spherical epithelial cells could be present, which is the regeneration phase of the vaginal epithelia, followed by ovulation (Fig. 5A). After ovulation, nucleated cornified epithelial cells could be observed in the estrus phase (Fig. 5A). As the epithelia degenerate, lymphocytes become more dominant in the smear, known as the met-estrus phase (Fig. 5A). In this phase, the same proportion among leukocytes, cornified, and nucleated epithelial cells could be present. This phase is followed by the total degradation of the vaginal epithelia in the resting part of the cycle, called the di-estrus phase. In this phase, the cytologic slide could be very poor in cells; just a few epithelial cells without a nucleus and some lymphocytes can be found (Fig. 5A). We selected the female rats for heart perfusion in the di-estrus phase, in which the estrogen and progesterone levels are considered the lowest [38].

### Ischemic preconditioning reduced the infarct size in both sexes irrespective of CKD

Infarct size was measured at week 9 to investigate the severity of I/R injury and the cardioprotective effect of IPRE in both sexes in CKD (Fig. 5B). Additionally, the coronary flow was measured 10 min after the cannulation of the hearts (coronary flow at 10 min [CF10’]), immediately after the global ischemia (CF80’), and at the end of the 2 h reperfusion (CF200’) to assess the ex vivo cardiac function (Figs. 1, 5C, and Additional file 1: Fig. S1A and 1B). The coronary flow at 10 min was similar in sham-operated and CKD animals of the same sex (Table 2), indicating no basal differences in the contractile function in response to CKD, which supports the echocardiographic results. Notably, CF10’ values were significantly lower in sham-operated and CKD females than in sham-operated and CKD males (63% and 70%, respectively) due to the smaller heart size of females vs. males (Table 2). The CF80’ values were significantly smaller in sham-operated females in the *I*/*R* subgroup than in the sham-operated males in the *I*/*R* subgroup due to the smaller heart size of females (^#^*p* = 0.049, Additional file 1: Fig. S1A). There was no significant difference in the CF80’ values between CKD males and females in the *I*/*R* subgroup (Additional file 1: Fig. S1A). However, CKD males in the *I*/*R* subgroups had significantly decreased CF80’ values compared to sham-operated males in the *I*/*R* subgroup (^†^*p* = 0.002, Additional file 1: Fig. S1A). Interestingly, IPRE significantly increased the CF80’ values in sham-operated and CKD males and females (to 150%, 163%, 135%, and 130%, respectively) (*p < 0.001, Additional file 1: Fig. S1A). The CF200’ values showed a similar pattern than the CF80’ values (Additional file 1: Fig. S1B). The only difference was that IPRE could not significantly increase the CF200’ value in the female CKD group as compared to the female sham-operated group (Additional file 1: Fig. S1B).

Similarly to the CF80’ values, IPRE significantly decreased infarct size in sham-operated and CKD males and females (to 60%, 55%, 55%, and 73%, respectively) (*p < 0.001, Fig. 5C); however, the presence of CKD did not significantly influence the size of infarction in either sexes (*p* = 0.294, Fig. 5C). Notably, the infarct size was significantly smaller in both sham-operated and CKD females in the *I*/*R* subgroups compared to sham-operated and CKD males in the *I*/*R* subgroups (^#^*p* < 0.001, 68% and 58%, respectively), indicating a marked cardioprotective effect of the female sex irrespective of CKD (Fig. 5C).

### The potential role of the SAFE and RISK pathways in the cardioprotective effect of IPRE in CKD in both sexes

**Fig. 6:**
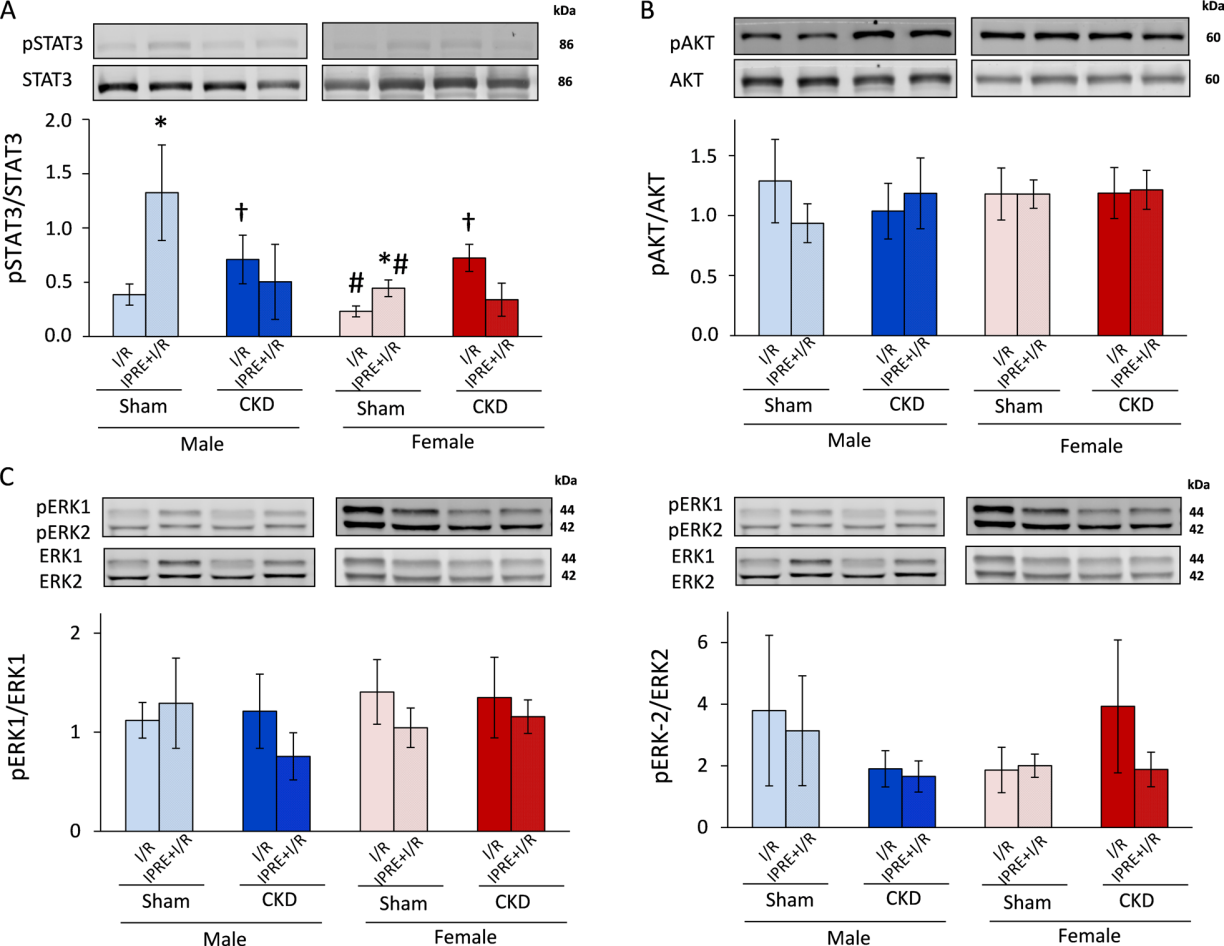
The effects of sex and CKD on the activation by phosphorylation of STAT3, AKT, and ERK1/2. **A** Phospho-STAT3/STAT3 ratios; **B** phospho-AKT/AKT ratios; **C** phospho-ERK1/ERK1 ratios; **D** phospho-ERK2/ERK2 ratios. Values are means ± SEM, *n* = 5–7 (male sham I/R: *n* = 5, male sham IPRE + I/R: *n* = 6, male CKD I/R: *n* = 6, male CKD IPRE + I/R: *n* = 5, female sham I/R: *n* = 6, female sham IPRE + I/R: *n* = 7, female CKD I/R: *n* = 7, and female CKD IPRE + I/R: *n* = 5), *p < 0.05, IPRE + I/R vs. I/R subgroups, #*p* < 0.05, females vs. males, ^†^*p* < 0.05, CKD vs. sham-operated groups, *p*-values refer to three-way ANOVA (Holm–Sidak post hoc test). *CKD* chronic kidney disease, *IPRE* ischemic preconditioning, I/R ischemia/reperfusion. Bands on the representative Western blot images correspond to the groups represented by bar graphs: 1. male sham I/R, 2. male sham IPRE + I/R, 3. male CKD I/R, 4. male CKD IPRE + I/R, 5. female sham I/R, 6. female sham IPRE + I/R, 7. female CKD I/R, 8. female CKD IPRE + I/R. Representative bands are framed on the original and uncropped images in Additional file 2: Fig. S2, Additional file 3: Fig. S3, Additional file 4: Fig. S4

At the end of reperfusion, a subgroup of hearts was collected for molecular measurements at the protein level (Fig. 6, Additional file 2: Fig. S2, Additional file 3: Fig. S3, Additional file 4: Fig. S4, Additional file 5: Fig. S5, Additional file 6: Fig. S6, Additional file 7: Fig. S7, Additional file 8: Fig. S8). Total proteins and their phosphorylated forms related to the cardioprotective SAFE (STAT3) and RISK (AKT, ERK1,2) pathways were measured by Western blot (Fig. 6, and Additional file 2: Fig. S2, Additional file 3: Fig. S3, Additional file 4: Fig. S4, Additional file 5: Fig. S5, Additional file 6: Fig. S6, Additional file 7: Fig. S7). IPRE significantly increased the phospho-STAT3/STAT3 ratio in sham-operated males and females compared to sex-matched sham-operated *I*/*R* subgroups, respectively (*p = 0.004, Fig. 6A). IPRE failed to significantly change the phospho-STAT3/STAT3 in CKD males and females compared to sex-matched CKD I/R subgroups, respectively (*p* = 0.094, Fig. 6A). In the female sham-operated *I*/*R* and preconditioned subgroups, the phospho-STAT3/STAT3 ratio was significantly lower than in the sham-operated male *I*/*R* and preconditioned subgroups, respectively (^#^*p* = 0.026, Fig. 6A). CKD abolished these lowering effects of the female sex on the phospho-STAT3/STAT3 ratio in the *I*/*R* and preconditioned subgroups compared to male *I*/*R* and preconditioned CKD subgroups, respectively (*p* = 0.164, Fig. 6A). Interestingly, the phospho-STAT3/STAT3 ratio was significantly higher in CKD males and females in the *I*/*R* subgroups compared to the sham-operated sex-matched *I*/*R* subgroups, respectively (^†^*p* = 0.025, Fig. 6A). This increasing effect of CKD on the phospho-STAT3/STAT3 ratio was not detectable in the preconditioned hearts as compared to sham-operated preconditioned subgroups in both sexes (*p* = 0.154, Fig. 6A). In phosho-AKT/AKT and phospho-ERK1,2/ERK1,2 ratios, there were no significant differences between the groups (Fig. 6B-6D).

## Discussion

We have found here that, although 9 weeks of experimental CKD leads to severe changes in metabolic parameters as well as myocardial morphology and function, the cardioprotective effect of ischemic preconditioning is preserved in both sexes. This is the first demonstration that CKD does not interfere with the cardioprotective effect of ischemic preconditioning in the female sex.

5/6 nephrectomy is probably the most established model of progressive renal failure mimicking the consequences of the reduction of functional nephron number [39, 40]. Our present findings are consistent with the literature data and our previous results in male 5/6 nephrectomy-induced CKD rats [28, 29, 39]. Here, we have found characteristic laboratory signs of CKD, including increased serum urea and creatinine concentrations and urine protein levels and diminished creatinine clearance with uremic cachexia in both sexes. Based on serum urea and creatinine levels as well as creatinine clearance normalized to percent changes, male and female rats developed CKD of similar severity 9 weeks after 5/6 nephrectomy. Among the routine laboratory parameters of CKD, only proteinuria was more severe in males compared to females in our present study, which is consistent with the findings of Lemos et al*.* using 5/6 nephrectomy in male and female Wistar rats to induce CKD [41]. Serum cholesterol levels were significantly higher in both sexes, pointing out the role of CKD in increased cardiovascular risk in both sexes [10]. Interestingly, serum triglyceride levels were significantly lower in females than in males independently of CKD, suggesting the cardiovascular risk lowering effect of the female sex; however, the precise molecular mechanism is yet unknown.

Both the Framingham Heart Study and Multi-Ethnic Study of Atherosclerosis demonstrated that left ventricular mass and volume are significantly greater in healthy men compared to women, even after adjusting for height and body surface area [42, 43]. These sex-based differences in the heart size are clearly seen in our present study in the heart weight, left ventricular weight, several wall thicknesses including anterior and septal wall thicknesses, left ventricular end-systolic diameter and cardiac output in the sham-operated animals. Premenopausal status in women is strongly related to better diastolic function and postmenopausal status is associated with accelerated age-related impairments in left ventricular relaxation [43]. Indeed, estrogen is described as a direct vasodilator and it could promote nitric oxide excretion, and directly improve myocardial calcium ion handling. All of these factors could improve the diastolic function in females [43]. In accordance with the aforementioned clinical findings and potential mechanisms, the diastolic parameters e’ and E velocities were significantly higher in females than in males in the sham-operated group in our present study.

In CKD patients, uremic cardiomyopathy is a common complication and reported to be a prognostic factor of cardiovascular mortality [10]. In our CKD model, marked cardiac morphological and functional changes were confirmed by echocardiography 8 weeks after the 5/6 nephrectomy. In our present study, male animals developed LVH with diastolic dysfunction and fibrosis as signs of uremic cardiomyopathy. We and others have previously shown that male CKD rats developed cardiac hypertrophy and fibrosis 9 or 12 weeks after 5/6 nephrectomy [29, 44, 45] which is consistent with our present results in male rats. However, there are only a very limited number of studies in the literature comparing the severity of cardiac hypertrophy and fibrosis between males and females in CKD induced by 5/6 nephrectomy [45, 46]. In the study of Paterson et al*.*, there was no difference in the left ventricular collagen content between sham-operated and 5/6 nephrectomy-induced CKD groups in female rats 7 weeks after the operations [45]. However, cardiac fibrosis showed a trend toward a decrease in the female CKD group when compared to the male CKD group in their study [45]. These results of Paterson et al. are similar to our present findings on statistically significant sex-based differences in cardiac fibrosis in CKD.

Severe hypertension is not usually a feature of the 5/6 nephrectomy-induced CKD models [39, 47]. Accordingly, the blood pressure was not significantly altered in CKD in either sex in our present study. Therefore, diastolic dysfunction might be developed as a direct consequence of LVH and cardiac fibrosis without severe hypertension in our CKD model. In our present study, female CKD rats developed a less severe LVH and diastolic dysfunction without cardiac fibrosis. An explanation for the less severe uremic cardiomyopathy in females could be the protective effects of estrogen via estrogen receptor-beta mediated anti-oxidative, anti-inflammatory and anti-apoptotic mechanisms [48, 49]. Further explanation could be that several genes related to adverse cardiac remodeling such as macrophage activation in the inflammatory processes, apoptosis, and lipid metabolism are located on the Y chromosome [48, 50]. Next to that, the quantity of viable cardiomyocytes in the healthy aging male heart decreases more rapidly than in the female heart, making the male heart already more prone to hypertrophy and stress [43, 48, 51]. In summary, our present echocardiographic and histologic findings on sex-based differences in the development of uremic cardiomyopathy are consistent with clinical observations that pre-menopausal women have a lower risk of developing LVH and cardiac remodeling [52].

To strengthen our echocardiographic and histologic results, we measured the left ventricular expression of ANP and BNP and the circulating BNP levels as myocardial stretch and heart failure markers [53]. ANP and BNP are released upon stimulation by stretching of the atrial and ventricular cardiomyocytes (e.g., overfilled heart), thereby causing natriuresis and diuresis, vasodilation and inhibition of the renin–angiotensin–aldosterone and sympathetic nervous system [54]. In our present study, left ventricular ANP expression was significantly increased in response to CKD independently of sex. In contrast, there was no significant difference between the groups in the left ventricular BNP expression and circulating BNP level. Notably, the circulating BNP level in females was half of the value of males in CKD suggesting a less severe cardiac remodeling in females than in males. These results are in accordance with our echocardiographic and histologic results.

The lack of severe hypertension and atherosclerosis in our CKD model makes it possible to study the direct effects of CKD on uremic cardiomyopathy and AMI. While traditional cardiovascular risk factors such as hypertension, atherosclerosis, and diabetes mellitus, are highly prevalent in CKD patients, results of clinical trials focusing on controlling these traditional cardiovascular risk factors have been mostly disappointing [39]. Both pre-clinical and clinical studies showed that CKD-specific risk factors such as uremic toxins, renal anemia, the over-activation of the renin–angiotensin–aldosterone system and sympathetic nervous system with increased oxidative/nitrative/nitrosative stress and decreased nitric oxide levels could provoke the development of uremic cardiomyopathy and increase the burden of AMI regardless of pressure- and volume overload [10].

CKD is a recognized risk multiplier for the development of CVDs [10], and AMI is a common cause of death in CKD [7]. A large body of evidence showed that cardiac conditioning strategies such as ischemic preconditioning, postconditioning, and remote conditioning are cardioprotective and significantly reduce myocardial I/R injury under experimental conditions [55]. Unfortunately, the clinical translation of cardioprotection by ischemic conditioning has been mostly disappointing. This unsuccessful clinical translation of ischemic conditioning strategies might be explained by the differences between pre-clinical models and the clinical scenario in AMI patients, including age, concomitant treatments, cardiovascular risk factors, and co-morbidities, such as aging, diabetes mellitus, and hyperlipidemia [56]. Moreover, unified recruitment strategies and algorithms of the conditioning stimulus, including the duration and number of *I*/*R* cycles, and its temporal distance to the index ischemia, are still lacking in the clinical phase trials [16].

Unfortunately, CKD patients are regularly excluded from clinical trials. However, we and others have previously shown that IPRE is cardioprotective in male rats in subacute and chronic renal failure, despite the complex changes in the systemic metabolism, including hypercholesterolemia and cardiac morphology and function [25, 28]. Therefore, CKD patients may also benefit from cardioprotection by ischemic conditioning strategies. This is particularly important since AMI frequently occurs in CKD patients. Interestingly, pre-clinical studies have shown that IPRE also remains cardioprotective in LVH [57] and hypertension [58, 59]. Therefore, we believe that the presence of LVH and diastolic dysfunction in CKD animals might not interfere with the cardioprotective effects of IPRE. However, it cannot be excluded that the cardioprotection by IPRE may be lost with the progression of CKD and the development of severe heart failure with reduced ejection fraction, as it has been reported that endogenous cardioprotection is lost in severe heart failure [24, 60, 61].

Epidemiological studies have shown that pre-menopausal women have a lower incidence of coronary artery disease than age-matched men [52, 62]. Pre-clinical studies have also shown that female animals have reduced *I*/*R* injury [63–66]. In this aspect, our infarct size results in females are in line with the literature data. However, due to the higher ischemic tolerance, the female sex is a potential confounder of ischemic conditioning interventions. Experimental studies dealing with this problem are sporadic and not conclusive, and clinical observations are mostly missing. In those limited number of studies comparing cardioprotection between both sexes, ischemic conditioning decreased the infarct size less in female than in male rat hearts [67, 68]. In our present study, the smaller reduction of the infarct size by ischemic conditioning in females compared to males is similar to the literature data. The menstrual cycle in female rats is short (4–5 days), and therefore several studies claimed that the acute fluctuation of estrogen and progesterone levels did not influence the myocardial ischemia/reperfusion injury [69]. In contrast, others showed that acute estrogen (i.e., 17β-estradiol) administration reduces the infarct size via reduction of mitophagy and the activation of the MEK/ERK/GSK-3β axis [70]. Therefore, we selected the female rats respecting the phase of their menstrual cycle before the perfusion protocols to eliminate the potential effects of fluctuating estrogen levels leading to inconclusive results in the infarct size.

In our current study, we also attempted to find possible downstream mechanisms of IPRE to relate the infarct size results with well-known cardioprotective mechanisms. Two major intracellular signaling pathways are considered as mediators of the IPRE stimulus, (i) the SAFE and (ii) RISK pathways [16]. STAT3 is considered a key component of the SAFE pathway, and AKT and ERK1,2 are believed to be the central mediators of the RISK pathway [16, 71]. We have assessed phosphorylation rates of STAT3, AKT, and ERK1,2 at the end of reperfusion, as infarct size analysis was performed at that time-point in our present study. We have shown here that 3 cycles of IPRE are associated with a significant increase in the phospho-STAT3/STAT3 ratio in the sham-operated animals 2 h after the end of the index ischemia in both sexes, which is in line with the literature data despite the late time-point [72]. In the sham-operated groups, IPRE failed to increase the phosphorylation of either AKT or ERK1,2 proteins significantly, respectively. This seems to be in contrast to some literature data and the general concept that the RISK pathway is a primary contributor to the cardioprotective effects of IPRE. This discrepancy might be explained by the experimental protocol that we used. We measured kinase phosphorylations 2 h after the end of the index ischemia. However, the phosphorylation status of IPRE-related kinases is mostly investigated at earlier time-points at the beginning of reperfusion. Nevertheless, several studies have already reported the later activation of cardiac SAFE and RISK pathways after 30, 120, or 180 min of reperfusion in response to ischemic conditionings [71, 73, 74].

In contrast with the findings by Byrne et al., the phospho-STAT3/STAT3 ratio failed to increase in male CKD rats in our present study. Our controversial result might be explained by the fact that Byrne et al. investigated the phosphorylation of STAT3 in an earlier and less severe phase of kidney disease and uremic cardiomyopathy (i.e., 4 weeks after the 5/6 nephrectomy). Another potential explanation is the previous activation of STAT3 in response to CKD in both sexes. Therefore, IPRE might be unable to increase the activation of STAT3 in CKD further. Notably, STAT3 is also a hypertrophy-inducing molecule [72], and LVH was confirmed in CKD in both sexes. Moreover, renal failure and the development of uremic cardiomyopathy also seem to interact with other protein kinases such as ERK1,2 and AKT, which activations are suggested to be involved in the mechanism of IPRE [29, 75, 76]. However, our present findings do not exclude the possible activation of the RISK and/or SAFE pathways by IPRE in earlier time-points of the reperfusion of the protocol that we used in sham-operated and/or CKD animals in both sexes.

### Limitations

Our study is not without limitations, similar to other experimental works. We have analyzed the infarct size and the SAFE and RISK pathways at a single time-point (i.e., 2 h after the end of the index ischemia). This fact might limit the proper interpretation of the early-phase molecular changes in the course of cardioprotection by IPRE. Therefore, investigation of the expression and activity of survival kinases in the SAFE and RISK pathways at earlier time-points might have some added value for our study. A common feature of experimental studies performed on *I*/*R* and conditioning strategies is that the ischemic cardiac tissue may contain viable and non-viable cells. The lack of separation of the viable and non-viable cells may affect the molecular markers in biochemical analyses leading to the blurring of results. The interpretation of these results could be a major problem, especially in studies using regional ischemia, because it is almost impossible to separate the non-ischemic, ischemic-viable, and ischemic-non-viable cells. Therefore, in our present study, we used global ischemia to expose all cardiac cells to the same ischemic stress. Despite these limitations, our study provides valuable data on the effect of experimental CKD on the infarct size-limiting effect and potential molecular mechanisms of ischemic preconditioning.

### Perspectives and significance

Taken together, males developed a more severe uremic cardiomyopathy in CKD than females. However, we have demonstrated for the first time in the present study, that the cardioprotective effect of IPRE is preserved in CKD in both sexes, even if CKD is associated with various structural, functional, and metabolic changes of the myocardium. The reason why the complex metabolic changes of CKD do not affect the overall efficacy of cardioprotection by IPRE is unknown. This result is exciting because several metabolic diseases, including diabetes mellitus and hyperlipidemia, abolish the endogenous cardioprotective mechanisms of ischemic conditioning strategies. Our present study suggests that CKD patients also benefit from cardioprotective strategies such as ischemic preconditioning regardless of their sex. Future pre-clinical studies investigating the molecular mechanisms of ischemic conditioning strategies in CKD in both sexes are needed. There is also an urgent need for clinical trials to investigate the cardioprotective effect of conditioning strategies in CKD patients of both sexes suffering from AMI.

## Supplementary Information

**Additional file 1: Fig. S1. **Coronary flow at the 80^th^ and 200^th^ minutes of the perfusion protocol. (A) coronary flow at the 80^th^ minute of the perfusion (CF80') and (B) coronary flow at the 200^th^ minute of the perfusion (CF200'). Values are means ± SEM, *n* = 15–18 in males (sham I/R: *n* = 17, sham IPRE + I/R: *n* = 18, CKD I/R: *n* = 15, and CKD IPRE + I/R: *n* = 16) and *n* = 15–28 in females (sham I/R: *n* = 15, sham IPRE + I/R: *n* = 15, CKD I/R: *n* = 27, and CKD IPRE + I/R: *n* = 28). *p < 0.05, IPRE + I/R vs. I/R subgroups, #*p* < 0.05, females vs. males, †*p* < 0.05, CKD vs. sham-operated groups, *p*-values refer to three-way ANOVA (Holm–Sidak post hoc test). CKD: chronic kidney disease, IPRE: ischemic preconditioning, I/R: ischemia/reperfusion.

**Additional file 2: Fig. S2. **Original uncropped and unmodified Western blot images. Representative bands used in Fig. 6 are framed.

**Additional file 3: Fig. S3. **Original uncropped and unmodified Western blot images. Representative bands used in Fig. 6 are framed.

**Additional file 4: Fig. S4. **Original uncropped and unmodified Western blot images. Representative bands used in Fig. 6 are framed.

**Additional file 5: Fig. S5. **Western blot results: phospho-STAT3/GAPDH and STAT3/GAPDH ratios. (A) Representative Western blot images, (B) phospho-STAT3/GAPDH ratios, (C) STAT3/GAPDH ratios. Values are means ± SEM, *n* = 5–7 (male sham I/R: *n* = 5, male sham IPRE + I/R: *n* = 6, male CKD I/R: *n* = 6, male CKD IPRE + I/R: *n* = 5, female sham I/R: *n* = 6, female sham IPRE + I/R: *n* = 7, female CKD I/R: *n* = 7, and female CKD IPRE + I/R: *n* = 5), *p < 0.05, CKD vs. sham-operated groups, #*p* < 0.05, females vs. males, *p*-values refer to three-way ANOVA (Holm–Sidak post hoc test). CKD: chronic kidney disease, IPRE: ischemic preconditioning, I/R: ischemia/reperfusion.

**Additional file 6: Fig. S6. **Western blot results: phospho-AKT/GAPDH and AKT/GAPDH ratios. (A) Representative Western blot images, (B) phospho-AKT/GAPDH ratios, (C) AKT/GAPDH ratios. Values are means ± SEM, *n* = 5–7 (male sham I/R: *n* = 5, male sham IPRE + I/R: *n* = 6, male CKD I/R: *n* = 6, male CKD IPRE + I/R: *n* = 5, female sham I/R: *n* = 6, female sham IPRE + I/R: *n* = 7, female CKD I/R: *n* = 7, and female CKD IPRE + I/R: *n* = 5), *p < 0.05, CKD vs. sham-operated groups, #*p* < 0.05, females vs. males, *p*-values refer to three-way ANOVA (Holm–Sidak post hoc test). CKD: chronic kidney disease, IPRE: ischemic preconditioning, I/R: ischemia/reperfusion.

**Additional file 7: Fig. S7. **Western blot results: phospho-ERK1/GAPDH and ERK1/GAPDH ratios. (A) Representative Western blot images, (B) phospho-ERK1/GAPDH ratios, (C) ERK1/GAPDH ratios. Values are means ± SEM, *n* = 5–7 (male sham I/R: *n* = 5, male sham IPRE + I/R: *n* = 6, male CKD I/R: *n* = 6, male CKD IPRE + I/R: *n* = 5, female sham I/R: *n* = 6, female sham IPRE + I/R: *n* = 7, female CKD I/R: *n* = 7, and female CKD IPRE + I/R: *n* = 5), *p < 0.05, CKD vs. sham-operated groups, #*p* < 0.05, females vs. males, *p*-values refer to three-way ANOVA (Holm–Sidak post hoc test). CKD: chronic kidney disease, IPRE: ischemic preconditioning, I/R: ischemia/reperfusion.

**Additional file 8: Fig. S8**. Western blot results: phospho-ERK2/GAPDH and ERK2/GAPDH ratios. (A) Representative Western blot images, (B) phospho-ERK2/GAPDH ratios, (C) ERK2/GAPDH ratios. Values are means ± SEM, *n* = 5–7 (male sham I/R: *n* = 5, male sham IPRE + I/R: *n* = 6, male CKD I/R: *n* = 6, male CKD IPRE + I/R: *n* = 5, female sham I/R: *n* = 6, female sham IPRE + I/R: *n* = 7, female CKD I/R: *n* = 7, and female CKD IPRE + I/R: *n* = 5), *p < 0.05, CKD vs. sham-operated groups, #*p* < 0.05, females vs. males, *p*-values refer to three-way ANOVA (Holm–Sidak post hoc test). CKD: chronic kidney disease, IPRE: ischemic preconditioning, I/R: ischemia/reperfusion.

## Data Availability

The datasets generated and analyzed during the current study are available from the corresponding author on a reasonable request.

## Abbreviations

AKT: Protein kinase B
AMI: Acute myocardial infarction
CKD: Chronic kidney disease
CVD: Cardiovascular disease
ERK1/2: Extracellular signal-regulated kinases 1 and 2
GSK-3β: Glycogen synthase kinase 3-beta
IPRE: Ischemic preconditioning
I/R: Ischemia/reperfusion
LV: Left ventricular
LVH: Left ventricular hypertrophy
MEK: Mitogen-activated protein kinase kinase
RISK: The reperfusion-induced salvage kinase
SAFE: Survivor activating factor enhancement
STAT3: Signal transducer and activator of transcription 3
