# Supplementary figures and images for: Ischemic preconditioning protects the heart against ischemia-reperfusion injury in chronic kidney disease in both males and females
Source: Biol Sex Differ. 2021 Sep 6;12:49. doi: 10.1186/s13293-021-00392-1 (PMC8420010; doi:10.1186/s13293-021-00392-1)

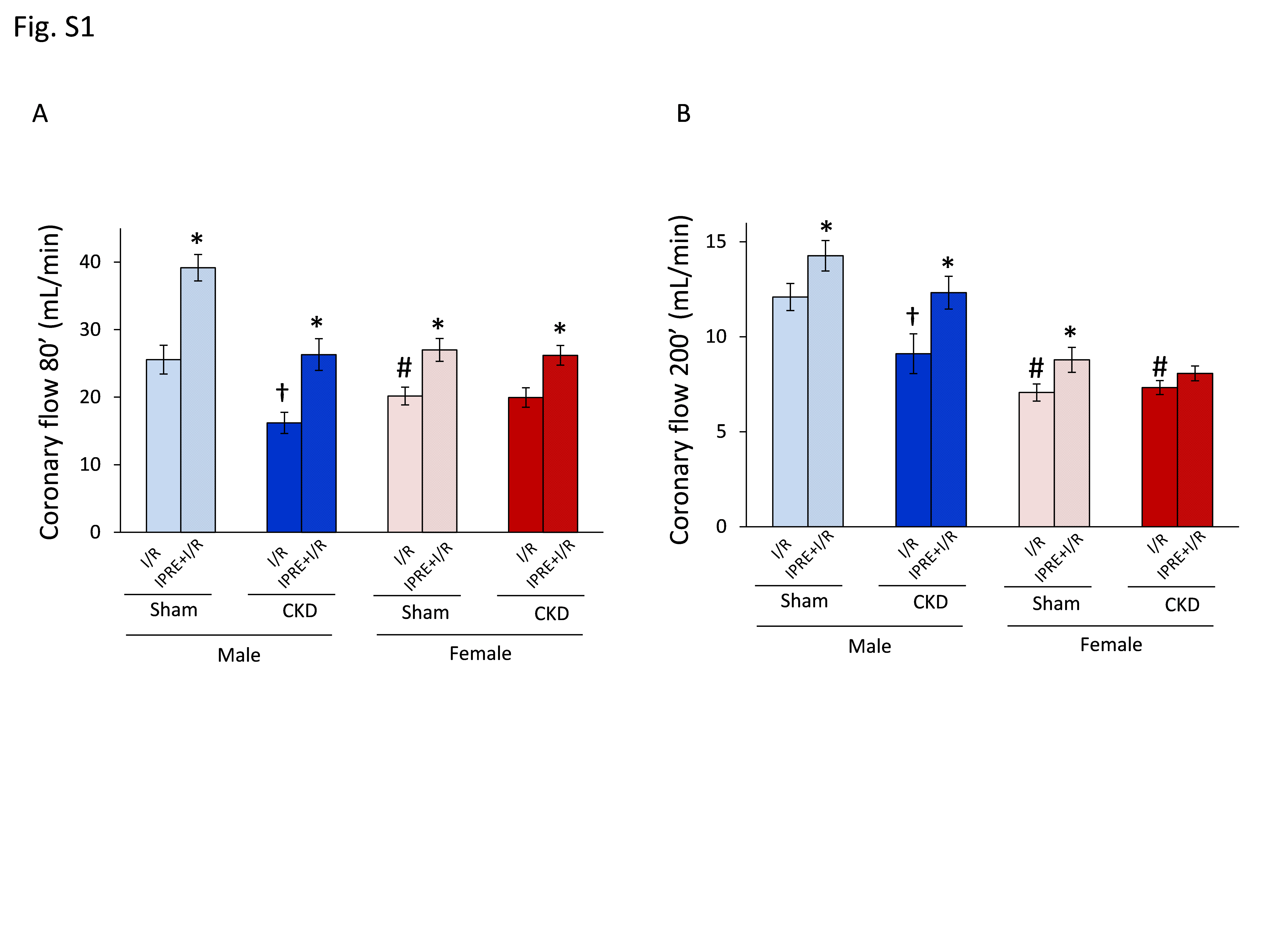

Supplement: Supplementary file 1 — Additional file 1: Fig. S1. Coronary flow at the 80th and 200th minutes of the perfusion protocol. (A) coronary flow at the 80th minute of the perfusion (CF80') and (B) coronary flow at the 200th minute of the perfusion (CF200'). Values are means ± SEM, n = 15–18 in males (sham I/R: n = 17, sham IPRE + I/R: n = 18, CKD I/R: n = 15, and CKD IPRE + I/R: n = 16) and n = 15–28 in females (sham I/R: n = 15, sham IPRE + I/R: n = 15, CKD I/R: n = 27, and CKD IPRE + I/R: n = 28). *p < 0.05, IPRE + I/R vs. I/R subgroups, #p < 0.05, females vs. males, †p < 0.05, CKD vs. sham-operated groups, p-values refer to three-way ANOVA (Holm–Sidak post hoc test). CKD: chronic kidney disease, IPRE: ischemic preconditioning, I/R: ischemia/reperfusion. [file 13293_2021_392_MOESM1_ESM.tif]

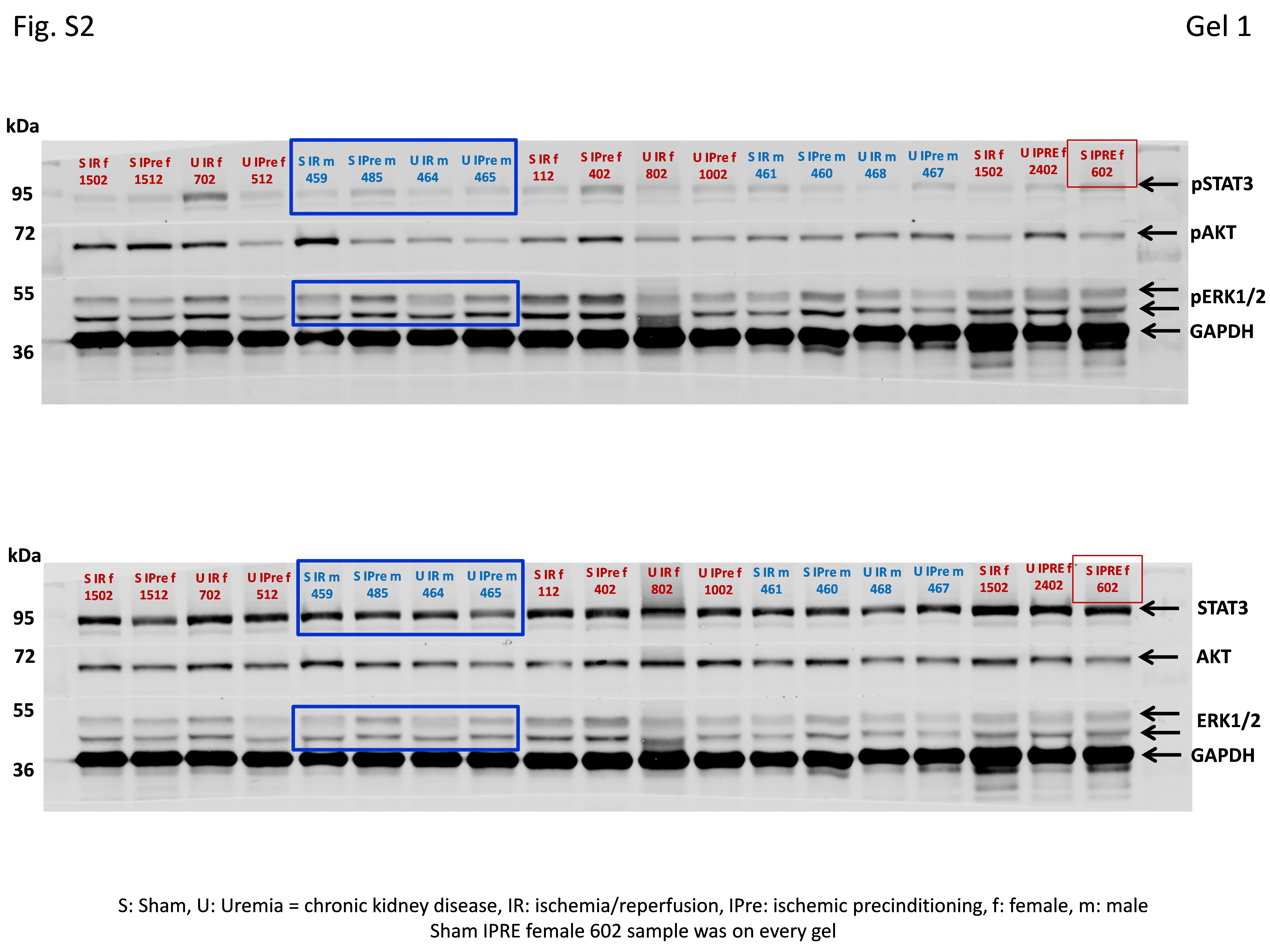

Supplement: Supplementary file 2 — Additional file 2: Fig. S2. Original uncropped and unmodified Western blot images. Representative bands used in Fig. 6 are framed. [file 13293_2021_392_MOESM2_ESM.tif]

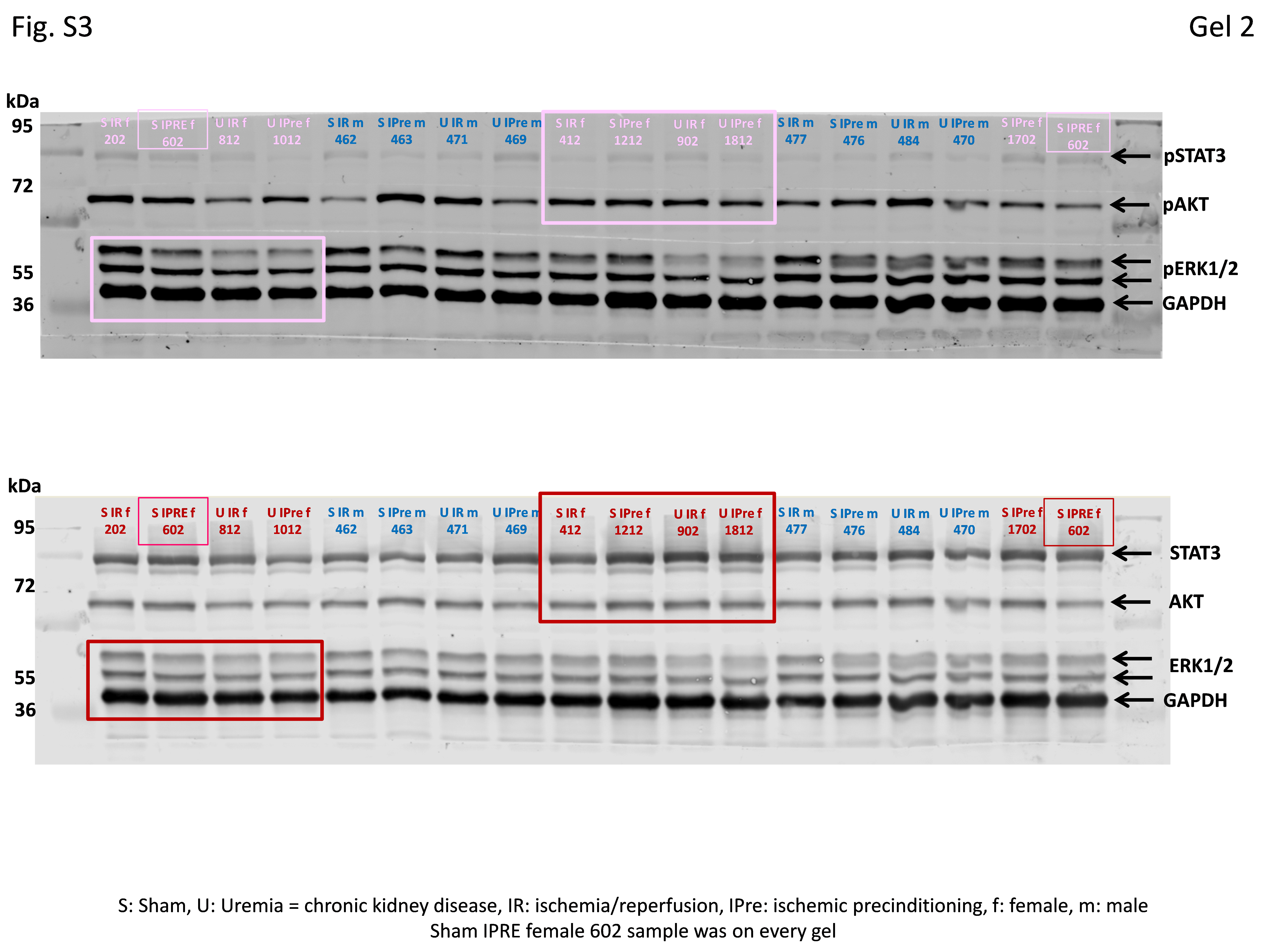

Supplement: Supplementary file 3 — Additional file 3: Fig. S3. Original uncropped and unmodified Western blot images. Representative bands used in Fig. 6 are framed. [file 13293_2021_392_MOESM3_ESM.tif]

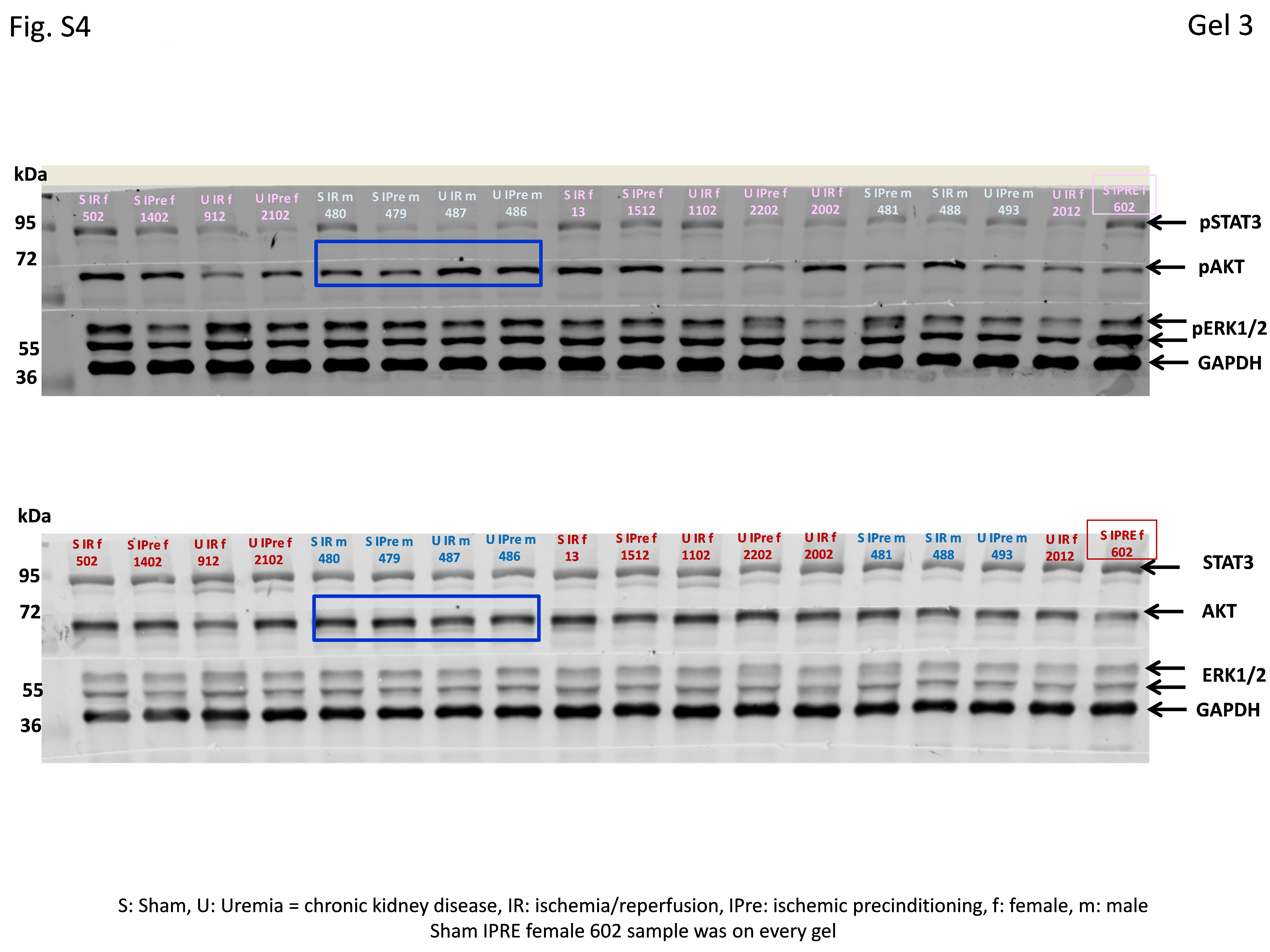

Supplement: Supplementary file 4 — Additional file 4: Fig. S4. Original uncropped and unmodified Western blot images. Representative bands used in Fig. 6 are framed. [file 13293_2021_392_MOESM4_ESM.tif]

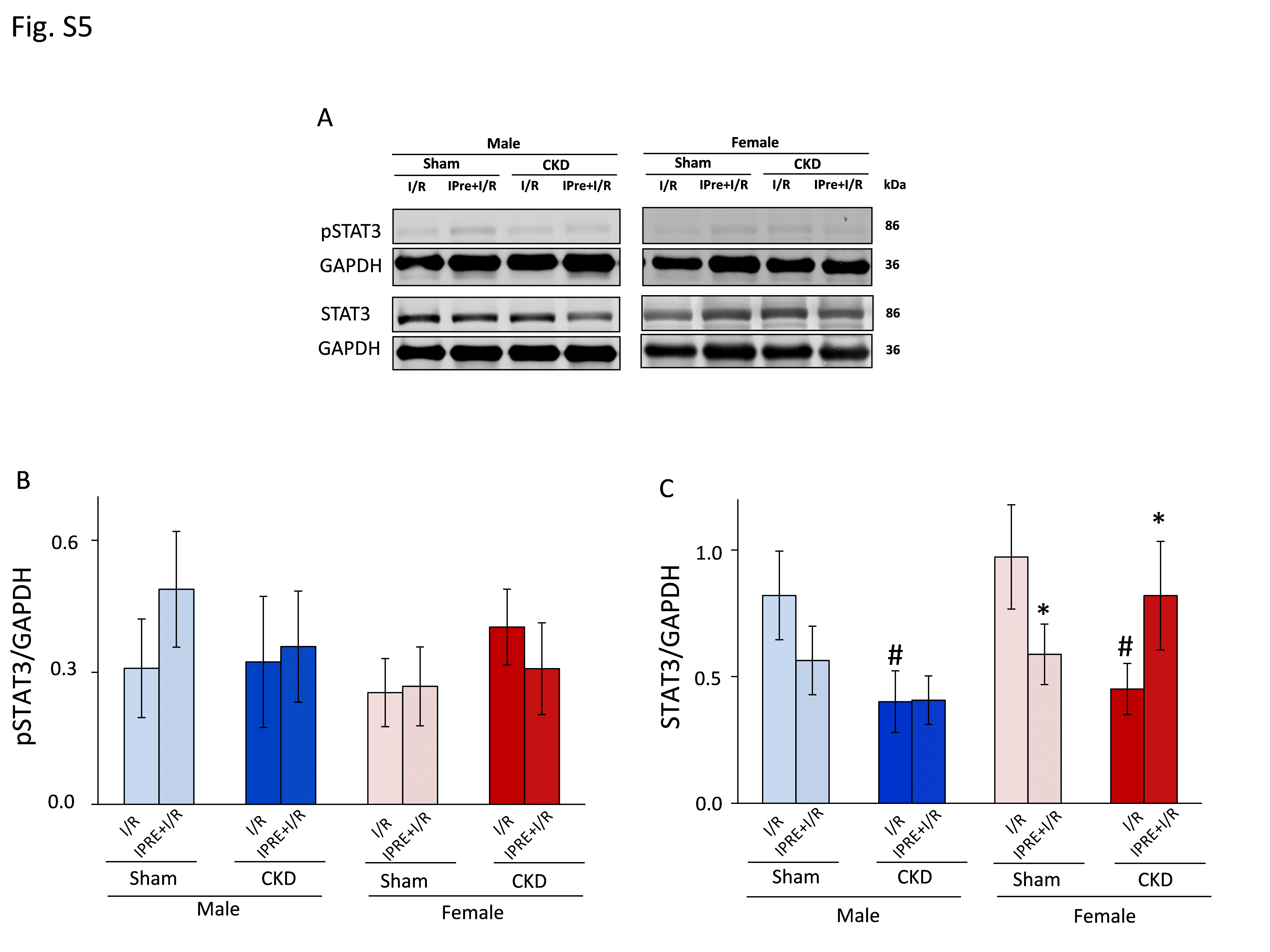

Supplement: Supplementary file 5 — Additional file 5: Fig. S5. Western blot results: phospho-STAT3/GAPDH and STAT3/GAPDH ratios. (A) Representative Western blot images, (B) phospho-STAT3/GAPDH ratios, (C) STAT3/GAPDH ratios. Values are means ± SEM, n = 5–7 (male sham I/R: n = 5, male sham IPRE + I/R: n = 6, male CKD I/R: n = 6, male CKD IPRE + I/R: n = 5, female sham I/R: n = 6, female sham IPRE + I/R: n = 7, female CKD I/R: n = 7, and female CKD IPRE + I/R: n = 5), *p < 0.05, CKD vs. sham-operated groups, #p < 0.05, females vs. males, p-values refer to three-way ANOVA (Holm–Sidak post hoc test). CKD: chronic kidney disease, IPRE: ischemic preconditioning, I/R: ischemia/reperfusion. [file 13293_2021_392_MOESM5_ESM.tif]

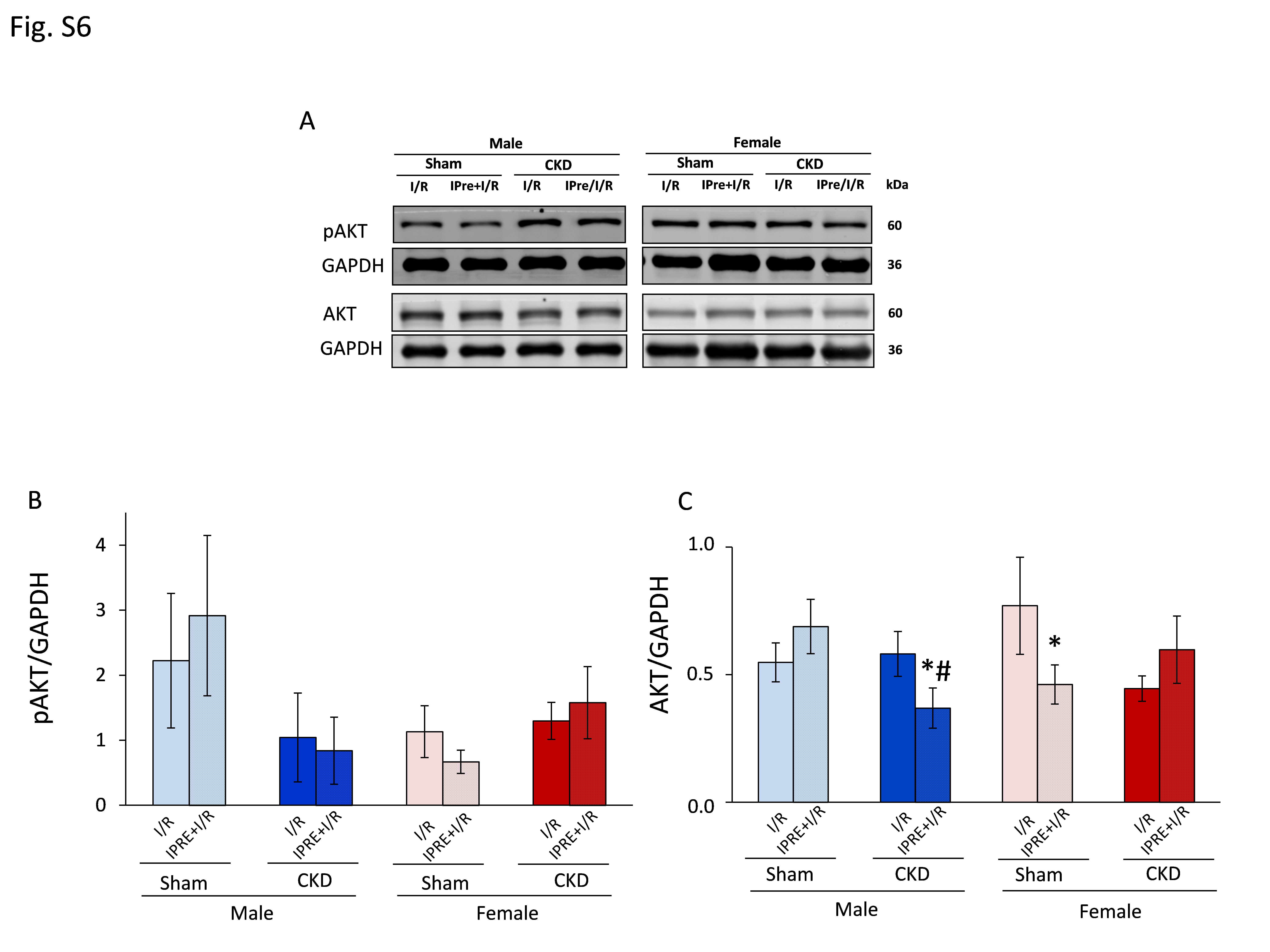

Supplement: Supplementary file 6 — Additional file 6: Fig. S6. Western blot results: phospho-AKT/GAPDH and AKT/GAPDH ratios. (A) Representative Western blot images, (B) phospho-AKT/GAPDH ratios, (C) AKT/GAPDH ratios. Values are means ± SEM, n = 5–7 (male sham I/R: n = 5, male sham IPRE + I/R: n = 6, male CKD I/R: n = 6, male CKD IPRE + I/R: n = 5, female sham I/R: n = 6, female sham IPRE + I/R: n = 7, female CKD I/R: n = 7, and female CKD IPRE + I/R: n = 5), *p < 0.05, CKD vs. sham-operated groups, #p < 0.05, females vs. males, p-values refer to three-way ANOVA (Holm–Sidak post hoc test). CKD: chronic kidney disease, IPRE: ischemic preconditioning, I/R: ischemia/reperfusion. [file 13293_2021_392_MOESM6_ESM.tif]

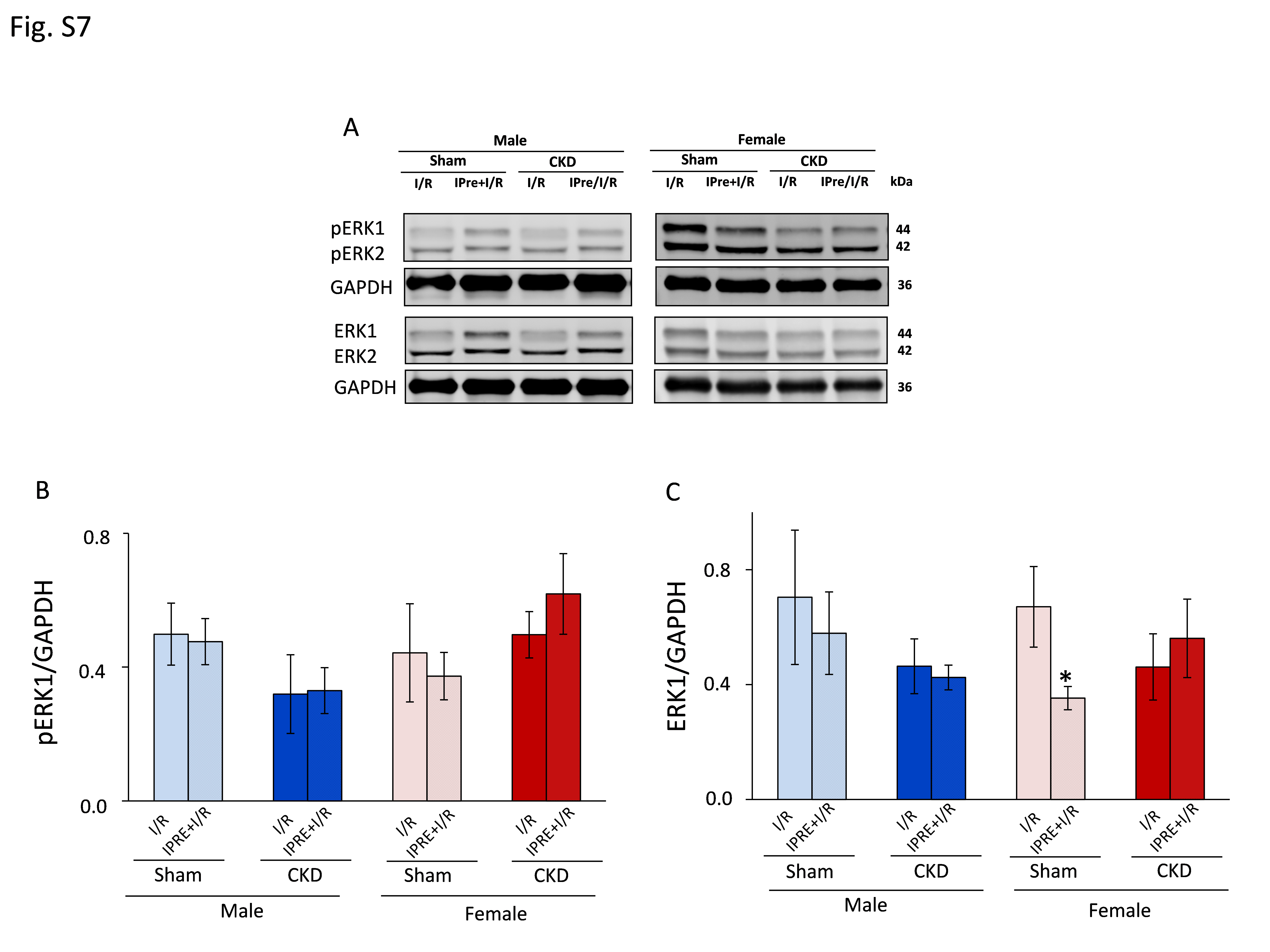

Supplement: Supplementary file 7 — Additional file 7: Fig. S7. Western blot results: phospho-ERK1/GAPDH and ERK1/GAPDH ratios. (A) Representative Western blot images, (B) phospho-ERK1/GAPDH ratios, (C) ERK1/GAPDH ratios. Values are means ± SEM, n = 5–7 (male sham I/R: n = 5, male sham IPRE + I/R: n = 6, male CKD I/R: n = 6, male CKD IPRE + I/R: n = 5, female sham I/R: n = 6, female sham IPRE + I/R: n = 7, female CKD I/R: n = 7, and female CKD IPRE + I/R: n = 5), *p < 0.05, CKD vs. sham-operated groups, #p < 0.05, females vs. males, p-values refer to three-way ANOVA (Holm–Sidak post hoc test). CKD: chronic kidney disease, IPRE: ischemic preconditioning, I/R: ischemia/reperfusion. [file 13293_2021_392_MOESM7_ESM.tif]

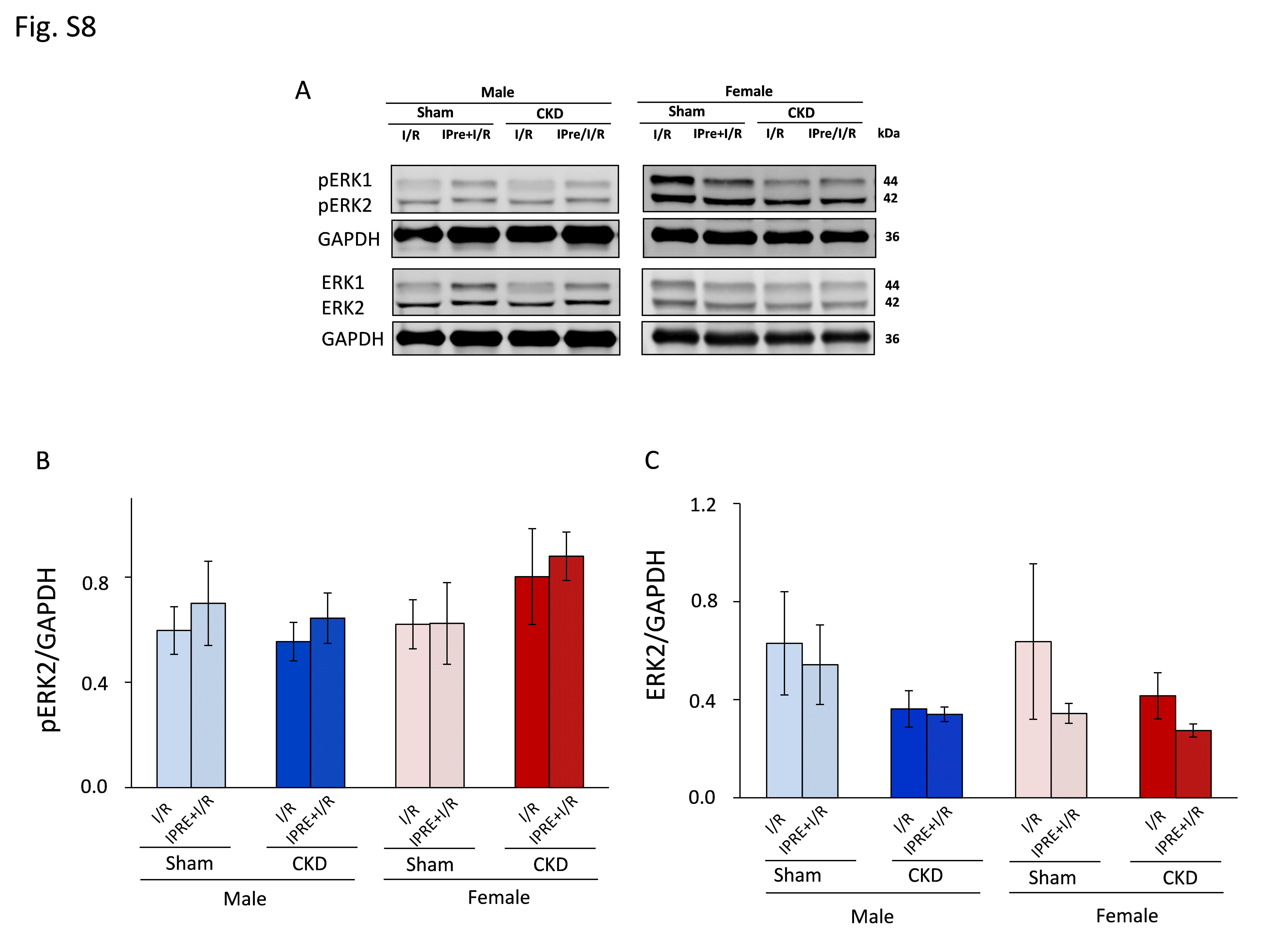

Supplement: Supplementary file 8 — Additional file 8: Fig. S8. Western blot results: phospho-ERK2/GAPDH and ERK2/GAPDH ratios. (A) Representative Western blot images, (B) phospho-ERK2/GAPDH ratios, (C) ERK2/GAPDH ratios. Values are means ± SEM, n = 5–7 (male sham I/R: n = 5, male sham IPRE + I/R: n = 6, male CKD I/R: n = 6, male CKD IPRE + I/R: n = 5, female sham I/R: n = 6, female sham IPRE + I/R: n = 7, female CKD I/R: n = 7, and female CKD IPRE + I/R: n = 5), *p < 0.05, CKD vs. sham-operated groups, #p < 0.05, females vs. males, p-values refer to three-way ANOVA (Holm–Sidak post hoc test). CKD: chronic kidney disease, IPRE: ischemic preconditioning, I/R: ischemia/reperfusion. [file 13293_2021_392_MOESM8_ESM.tif]
